# Environmental levels of microplastics disrupt growth and stress pathways in edible crops via species-specific mechanisms

**DOI:** 10.3389/fpls.2025.1670247

**Published:** 2025-08-28

**Authors:** Zhangling Chen, Laura J. Carter, Steven A. Banwart, Paul Kay

**Affiliations:** ^1^ School of Earth and Environment, University of Leeds, Leeds, United Kingdom; ^2^ School of Geography, University of Leeds, Leeds, United Kingdom

**Keywords:** microplastics, soil properties, plant physiology, ecotoxicology, oxidative stress, agricultural sustainability

## Abstract

**Introduction:**

Microplastics (MPs) are emerging contaminants in agricultural soils. However, the responses of different plant species to MP stress under soil conditions across varying concentration levels, as well as the underlying mechanisms, remain insufficiently understood.

**Methods:**

This study examined the morphological, physiological, and biochemical responses of Chinese cabbage (*Brassica rapa*) and cherry radish (*Raphanus sativus*) grown in soil containing polystyrene microplastics (PS-MPs) at concentrations of 10, 50, and 100 mg/kg.

**Results:**

PS-MPs altered soil properties by increasing pH and water-holding capacity (WHC), which promoted early germination in both species. However, during later growth stages, MPs inhibited development in a species-specific manner. In cherry radish, root length and fruit diameter decreased by 35.0% and 20.4%, respectively, primarily due to physical blockage. In Chinese cabbage, leaf area and petiole number declined by 35.9% and 41.7%, mainly driven by soil structural disruption and nutrient loss. Notably, the most pronounced effects occurred at low (10 mg/kg) to medium (50 mg/kg) concentrations, indicating a non-linear dose–response relationship. Hierarchical regression analysis (HRA) further revealed distinct toxicity mechanisms: physical accumulation and localized hypoxia were predominant in cherry radish, whereas oxidative stress and redox imbalance played a central role in Chinese cabbage.

**Discussion:**

These findings support current ecotoxicological models and highlight the importance of plant–particle interactions in shaping crop responses. The results provide new insight into MP phytotoxicity and inform future risk assessments under realistic soil conditions.

## Graphical Abstract

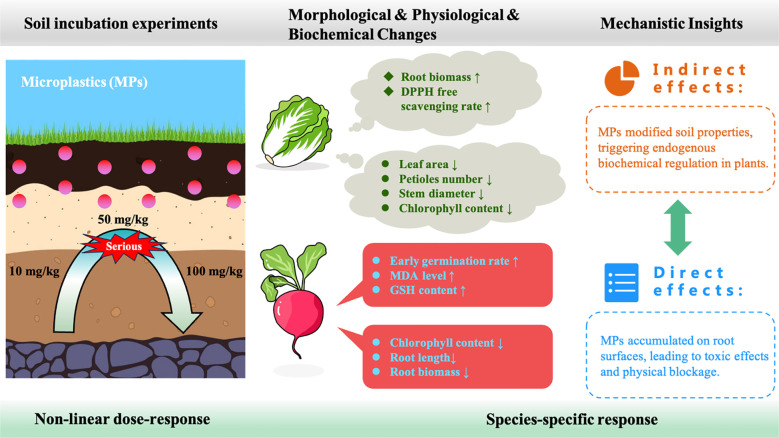

## 1 Introduction

The global production of plastics has increased rapidly in recent decades, reaching 413.8 million metric tons in 2023 (Statista, 2025). While plastics are valued for their durability and versatility, these same properties lead to their persistence in the environment. Microplastics (MPs), defined as particles smaller than 5 mm, result from the breakdown of larger plastic debris or are manufactured at microscopic scales (Thompson et al., 2004). Although research on MPs has primarily focused on aquatic ecosystems, terrestrial environments are now recognized as major MP sinks (Rillig and Lehmann, 2020; Chen et al., 2024). Around 80% of marine plastics originate from land-based sources, and MP concentrations in soil are estimated to be 4 to 23 times higher than in marine systems (Horton et al., 2017). Agricultural land, covering approximately 38% of the Earth’s surface, is particularly vulnerable to MP pollution due to practices such as plastic mulching, sewage sludge application, and irrigation with treated wastewater (Jin et al., 2022). These inputs can alter soil structure, disrupt microbial communities, and interfere with nutrient cycling (Wang et al., 2024; Han et al., 2024).

As primary producers in agroecosystems, crops are directly affected by MP contamination. In recent years, growing attention has been devoted to understanding how MPs influence plant growth, revealing complex and often inconsistent outcomes. MPs are known to inhibit seed germination by physically obstructing root elongation, as observed in lentil (*Lens culinaris*) (De Silva et al., 2022). However, contrasting findings have been reported; for example, rice (*Oryza sativa L.*) and cherry tomato (*Solanum lycopersicum L.*) showed no significant changes in germination under MP exposure (Zhang et al., 2021; Shorobi et al., 2023). In terms of vegetative growth, Qi et al. (2018) found that 1% w/w biodegradable plastics (Bio–MPs) exposure reduced wheat (*Triticum aestivum*) height, whereas Meng et al. (2021) observed increased root length in common bean (*Phaseolus vulgaris L.*) under similar treatment. Likewise, de Souza MaChado et al. (2019) reported that 0.2% w/w polyester (PES) microfibers decreased perennial ryegrass (*Lolium perenne*) biomass, while Lozano and Rillig (2020) found the same treatment enhanced spring onion (*Allium fistulosum*) biomass. Li et al. (2020b) showed that 0.5% w/w polyvinyl chloride (PVC) MPs increased average root diameter in lettuce (*Lactuca sativa L.*), whereas 2% w/w PVC-MPs reduced it. Oladele et al. (2023) further demonstrated that 0.5% w/w polystyrene (PS) MPs elevated chlorophyll content and total nitrogen (N) and phosphorus (P) accumulation in cowpea (*Vigna unguiculata*), while 4% w/w PS-MPs impaired soil enzyme activity and altered microbial communities. Collectively, these findings indicate that MP impacts on plants are highly species-specific and dose-dependent, with the magnitude and direction of responses differing significantly. They underscore that both exposure conditions and plant functional traits play critical roles in determining the ecological consequences of MP pollution.

However, most studies on the effects of MPs on plants have relied on hydroponic systems with acute exposure (Li et al., 2024; Xiao et al., 2024). Such approaches do not accurately reflect soil-based conditions, where MPs interact with organic matter, soil microbes, and plant root architecture, thereby influencing their mobility, bioavailability, and toxicity (Khalid et al., 2020; En-Nejmy et al., 2024; Rillig, 2020). Furthermore, MP concentrations used in many experiments often exceed environmentally relevant levels, while actual MP loads in agricultural soils vary considerably among regions due to differences in environmental conditions and socioeconomic factors (Chen et al., 2025). In addition, although multiple crop species commonly coexist in agricultural systems, the mechanisms underlying their species–specific responses to MP exposure remain insufficiently understood. Therefore, evaluating these responses under realistic soil conditions and across environmentally relevant concentration gradients is crucial for accurately assessing their ecological risks in agricultural ecosystems.

In this study, polystyrene microspheres (PS–MPs) were chosen as the model MPs because of their uniform size distribution, stable fluorescence, and widespread use in mechanistic and ecotoxicological research, which allows clear visualization of particle–plant interactions (Li et al., 2020a; Jiang et al., 2019; Rong et al., 2024). Although polyethylene (PE) is more commonly used in agricultural mulching films, PS particles are also present in compost, biosolids, personal care product residues, and degraded packaging waste, making them environmentally relevant in agricultural settings (Qiang et al., 2023; Ceccanti et al., 2024; Mathew et al., 2024). We investigated the effects of soil-applied PS–MPs on two edible crops—Chinese cabbage (*Brassica rapa*) and cherry radish (*Raphanus sativus*)—which differ in growth form and tissue type. A concentration gradient of PS–MPs (10, 50, and 100 mg/kg) was applied to represent environmentally relevant levels as well as a projected future scenario. Plant responses were evaluated across morphological, physiological, and biochemical dimensions, and hierarchical regression analysis (HRA) was employed to explore species-specific response mechanisms and dose-dependent toxicity patterns. This integrated approach provides new insights into how MPs influence plant development under realistic soil conditions and offers a framework for assessing their impacts on food production and agroecosystem sustainability.

## 2 Materials and methods

### 2.1 Microplastic selection and characterization

**Figure 1:**
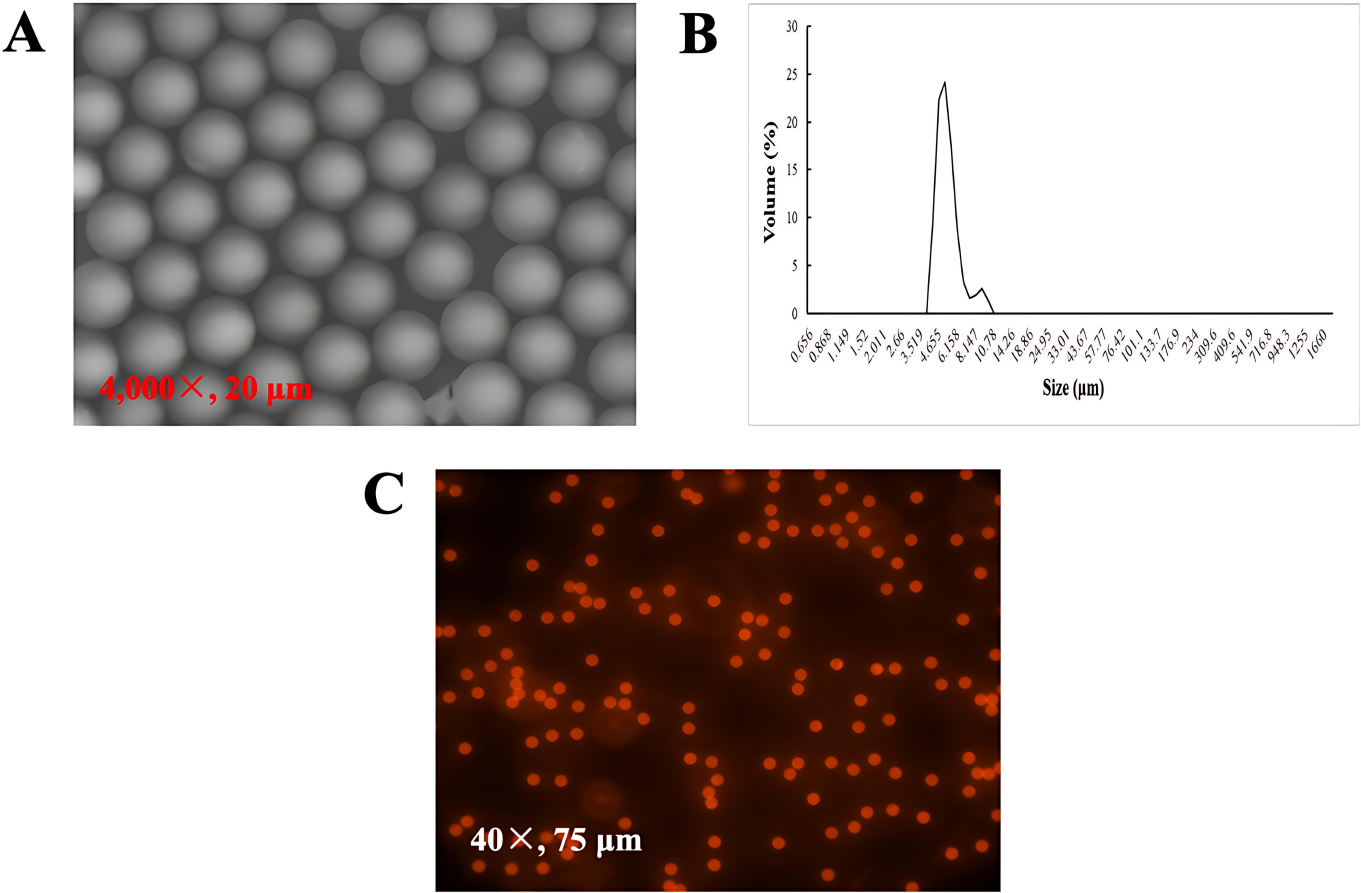
Characterization of 5 μm fluorescent PS-MPs. **(A)** SEM image of particle morphology; **(B)** Particle size distribution, determined using DLS; **(C)** Fluorescence images of the particles, captured using the EVOS FL Auto 2 system with an RFP light filter.

Red fluorescent PS-MPs were purchased from Jiangsu Zhichuan Technology Co., Ltd. (China) and suspended in deionized water at an initial concentration of 25 mg/mL. Their morphology was analyzed using a scanning electron microscope (SEM) (**Figure 1A**), while size distribution was measured through dynamic light scattering (DLS) analysis (**Figure 1B**). The particles exhibited a mean diameter of 5.01 ± 0.05 μm and a surface charge of -35 mV. Fluorescent imaging, conducted with the EVOS FL Auto 2 system, confirmed the uniform particle size and absence of fluorescent dye leakage (**Figure 1C**).

### 2.2 Soil preparation

Kettering loam soil was chosen as the substrate due to its uniform texture, high nutrient retention capacity, and common application in agricultural research (Lozano et al., 2021). The soil was sourced in 25 kg bags from Pitchcare (United Kingdom) and had undergone standard sterilization to eliminate harmful pathogens. The soil properties include a pH of 6.8, organic carbon content of 2.5%, and cation exchange capacity of 18 cmol/kg. Before use, the soil was sieved through a 2 mm mesh, air-dried, and homogenized. PS-MPs were manually mixed into the soil in a tray to achieve uniform distribution.

### 2.3 Experimental setup

Chinese cabbage (*Brassica rapa*) and cherry radish (*Raphanus sativus*) were selected as model edible crops due to their similar growth requirements in greenhouse conditions, facilitating consistent experimental setups. Additionally, these crops represent leafy and root vegetables, respectively, offering insights into species-specific responses to MP contamination. Seeds were procured from Mr. Fothergill’s (a commercial seed supplier, United Kingdom) and sterilized in 10% sodium hypochlorite (NaClO) for five minutes, followed by three rinses with deionized water to remove residual chemicals. The seeds were then dried with filter paper before sowing.

Three seeds were sown in each glass jar (8.5 cm in diameter, 9.4 cm in height), and after the seedlings developed two true leaves, thinning was conducted to retain only the most vigorous seedling per jar. PS-MP concentrations of 10 mg/kg (low), 50 mg/kg (medium), and 100 mg/kg (high) were selected. Low and medium concentrations align with MP levels detected in agricultural soils (Büks and Kaupenjohann, 2020), while the high concentration corresponds to projections from a Poisson regression model predicting that MP levels in agricultural soils could reach 168.9 mg/kg within 50 years (Meizoso-Regueira et al., 2024). These concentrations thus simulate both present-day and near-future scenarios, and a control group with no PS-MPs (0 mg/kg) was included for comparison.

The experiment consisted of two consecutive 50-day soil incubation periods, covering the full life cycle of the plants, and was conducted in a greenhouse under controlled conditions (12:12 h light/dark cycle at 19 °C). Jar positions within the greenhouse were randomly assigned using a random number table, and jars were rotated weekly to minimize positional bias caused by potential spatial variation in light and temperature. Because the glass jars did not have drainage holes in order to prevent the loss of PS-MPs with leachate, a pilot test was conducted to determine an appropriate irrigation regime. Soil moisture was maintained at approximately 65 ± 5% of field capacity by daily irrigation with deionized water, which avoided waterlogging and ensured normal root development. All plants were harvested at the end of the incubation period, and each treatment consisted of five biologically independent replicates (one plant per jar) (**Supplementary Figure S1**).

### 2.4 Soil physical and chemical properties measurement

Soil pH was measured by mixing 10 g of air-dried soil with deionized water. Bulk density was determined using professional tins (8 cm in diameter, 5.2 cm in height), with soil samples oven-dried at 105°C for 48 hours. Water-holding capacity (WHC) and water-stable aggregates (WSA) were assessed following established protocols detailed in the **Supplementary Material** (**Supplementary Texts S1, S2**). Total organic carbon (TOC) and total nitrogen (TN) contents were quantified via high-temperature combustion using an Analytik Jena Multi NC2100S instrument equipped with an NDIR detector. For measurements, 10 mg of finely ground soil (<100 μm) was treated with 30 μL of 15% hydrochloric acid (HCl) to remove inorganic carbon. The samples were then dried at 80°C for 24 hours and encapsulated in silver capsules prior to analysis.

### 2.5 Microscopy observation

The EVOS FL Auto 2 imaging system was used to visualize the accumulation of PS-MPs in Chinese cabbage and cherry radish. For sample preparation, plant roots were meticulously sectioned with a sharp scalpel and placed in Petri dishes containing a few drops of deionized water to remove residual soil while preserving hydration. Images were acquired at 40× magnification, equipped with RFP and Trans light cubes.

### 2.6 Plant morphological and physiological endpoints measurement

The overall germination rate was recorded daily from sowing to the seventh day. At 50 days post-sowing, plants were carefully removed from glass jars using a spatula. Roots were excised with a scalpel, washed with deionized water to remove soil residues, and dried with paper towels. The number of petioles was counted, and plant samples were positioned alongside a calibrated ruler on a sterilized bench for high-resolution imaging. Morphological endpoints, including root length, stem (fruit) diameter, and leaf area, were quantified from these images using ImageJ software (version 1.54g, **Supplementary Text S3**).

For biomass determination, roots were dried at 60°C for 72 hours (Lozano and Rillig, 2020). Chlorophyll content was extracted using 90% acetone, and the absorbance of the supernatant was measured at 664 nm and 647 nm using a Jasco Scanning Spectrophotometer. Chlorophyll a (Chla), chlorophyll b (Chlb), total chlorophyll content, and chlorophyll ratios were calculated based on equations provided in the **Supplementary Material** (**Supplementary Text S4**).

### 2.7 Oxidative stress biomarkers selection and assays

Malondialdehyde (MDA), superoxide dismutase (SOD), reduced glutathione (GSH), and the 2,2–diphenyl–1–picrylhydrazyl (DPPH) free radical scavenging rate were selected as key biomarkers to evaluate oxidative stress. These indicators collectively assess lipid peroxidation, oxidative defense mechanisms, and the antioxidative capacities of plants under MP stress. All assays were conducted using commercial kits from Nanjing Jiancheng Bioengineering Institute (China), following the manufacturer’s protocols detailed in the **Supplementary Material** (**Supplementary Texts S5–S8**).

### 2.8 Statistical analysis

A one-way analysis of variance (ANOVA) followed by Duncan’s *post hoc* test was performed to compare differences among treatment groups, with separate analyses conducted for each plant species (Chinese cabbage and cherry radish) across the four PS–MP concentration levels (0, 10, 50, and 100 mg/kg). Prior to ANOVA, data normality was assessed using the Shapiro–Wilk test, and homogeneity of variances was evaluated using Levene’s test.

To further explore species-specific response mechanisms under MP stress, we employed hierarchical regression analysis (HRA), which enables stepwise inclusion of variables to separate the direct effects of PS–MP concentration from those mediated through biochemical regulation. This approach provides a quantitative and mechanistic basis for linking observed phenotypes with underlying stress mechanisms and offers deeper insight than simple regression or correlation analysis. HRA followed a two-step modeling framework: the baseline model (R_1_²) evaluated the influence of PS–MP concentration on plant growth traits, while the extended model (R_2_²) incorporated biochemical indicators to capture additional variance. The change in explained variance (ΔR² = R_2_² − R_1_²) was interpreted as the extent to which biochemical processes mediate plant responses to MP exposure. For instance, a ΔR² value of 0.50 indicates that biochemical regulation accounts for half of the explained variation in plant growth traits, underscoring its dominant role in modulating responses under MP stress.

All statistical analyses were conducted using IBM SPSS Statistics (version 27), with significance set at *p*<0.05.

## 3 Results and discussion

### 3.1 Germination and morphological alterations in Chinese cabbage and cherry radish under PS-MP exposure

**Figure 2:**
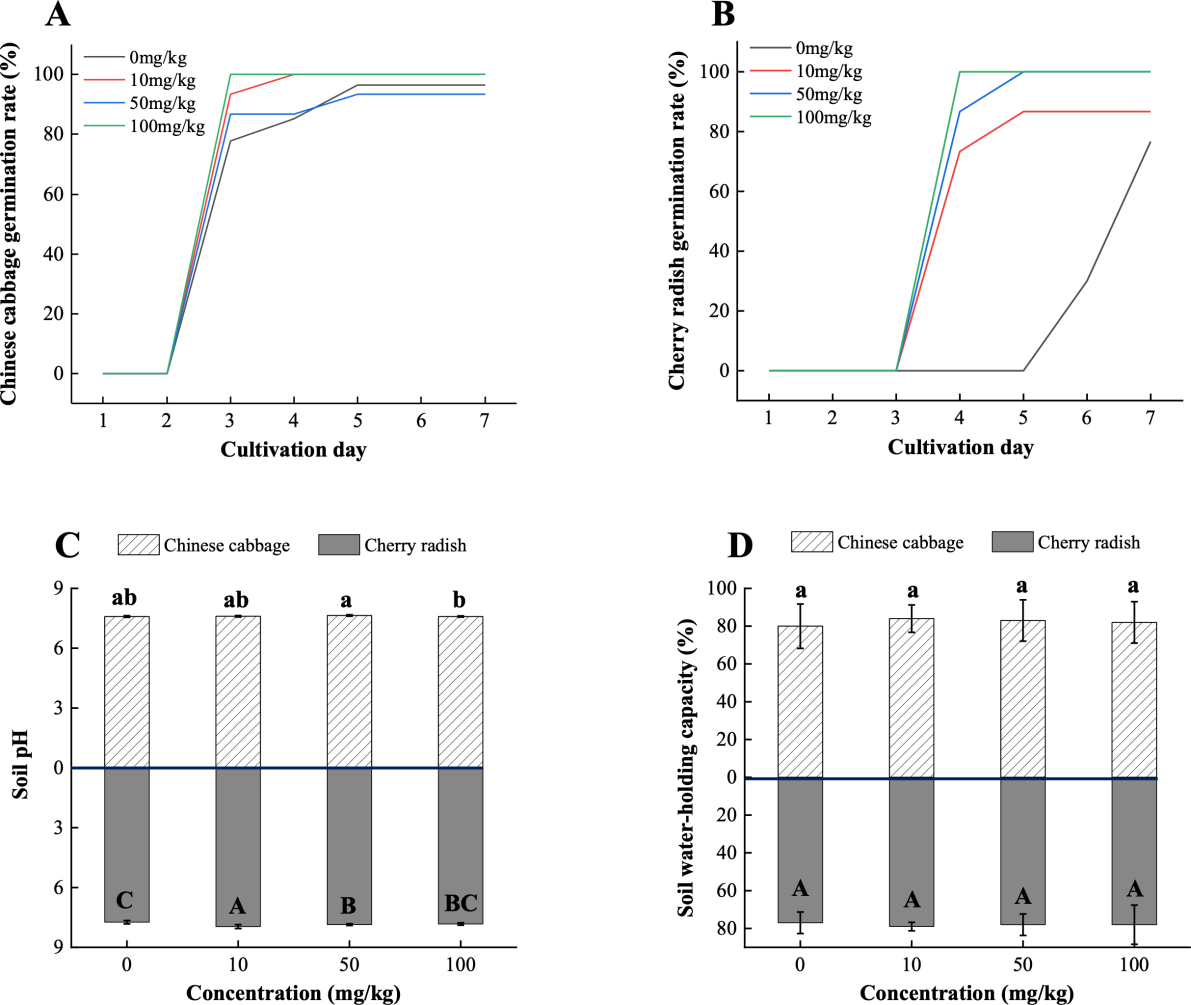
Overall germination rates and soil properties of two edible plants under PS-MP treatments. **(A)** Overall germination rate of Chinese cabbage; **(B)** Overall germination rate of cherry radish; **(C)** pH values in soils from two crop species; **(D)** Water-holding capacity of soils from two crop species. [1] Results are presented as mean ± SD (Standard Deviation), N= 5. [2] Letter labels (e.g., a, b, ab/A, B, AB) are assigned using Duncan's post hoc test to indicate significant group differences. Groups sharing the same letter are not significantly different, while groups with different letters exhibit significant differences. [3] Statistical analyses are performed separately for Chinese cabbage and cherry radish, so uppercase and lowercase letter groupings represent independent tests for each species.

The presence of PS-MPs accelerated the overall germination of both Chinese cabbage and cherry radish. At 100 mg/kg PS-MPs, cherry radish seeds achieved 100% germination by the fourth day, significantly outpacing the control group (0%; **Figure 2B**). Similarly, Chinese cabbage in the 100 mg/kg group reached a 100% germination rate by the third day, compared to 77.8% in the control (**Figure 2A**). This unexpected enhancement in germination could be attributed to the following mechanisms: (1) Changes in soil pH. As shown in **Figure 2C**, the addition of PS-MPs significantly increased soil pH (*p* < 0.05). This effect was consistent across soils cultivated with both Chinese cabbage and cherry radish and was independent of MP concentration gradients. A slightly elevated pH may alleviate soil acid stress and enhance ion exchange capacity, thereby creating a more favorable environment for seed germination (Boots et al., 2019). Changes in soil pH can influence the solubility and availability of essential nutrients, such as calcium (Ca) and magnesium (Mg) ions, which are important for plant early growth stages (Follmer et al., 2021). Furthermore, the surface properties of MPs, including hydrophobicity and electrostatic interactions, may further modulate these processes by altering the ionic balance in the soil matrix (Rico et al., 2011). (2) Improvement in soil WHC. An increase in WHC was observed in soils cultivated with both Chinese cabbage and cherry radish (*p* > 0.05; **Figure 2D**). Enhanced WHC could provide a more consistent water supply during the germination phase, enabling seeds to absorb sufficient moisture. This phenomenon aligns with findings by de Souza MaChado et al. (2019), which suggest that MPs can alter soil properties, potentially improving soil moisture retention. These modifications in soil properties likely synergize to support improved conditions for seed hydration and nutrient dynamics, thereby facilitating germination and promoting early growth.

**Figure 3:**
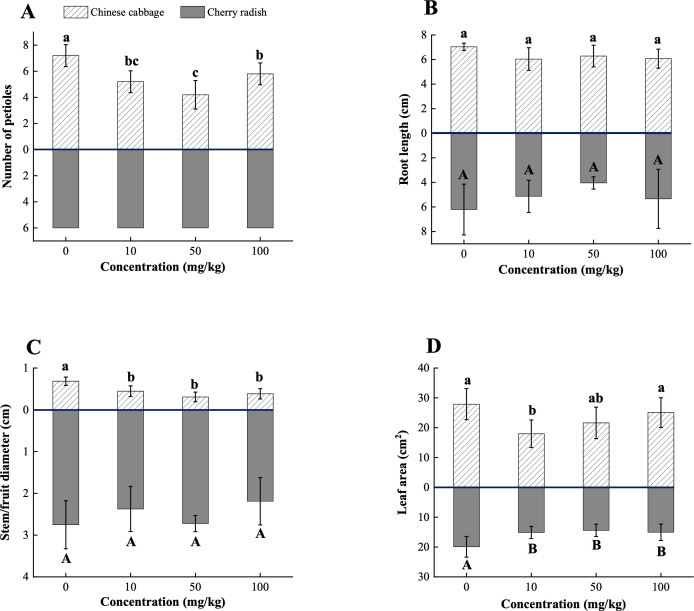
Morphological traits of two edible plants under PS-MP treatments. **(A)** Number of petioles; **(B)** Root length; **(C)** Stem/fruit diameter; **(D)** Leaf area. [1] Results are presented as mean ± SD (Standard Deviation), N= 5. [2] The number of petioles in cherry radish (**Figure 3A**, grey columns), which shows no variability across treatments, resulting in zero standard deviation and the absence of error bars. [3] Letter labels (e.g., a, b, ab/A, B, AB) are assigned using Duncan's post hoc test to indicate significant group differences. Groups sharing the same letter are not significantly different, while groups with different letters exhibit significant differences. [4] Statistical analyses are performed separately for Chinese cabbage and cherry radish, so uppercase and lowercase letter groupings represent independent tests for each species.

PS-MPs significantly reduced petiole number and stem diameter in Chinese cabbage (*p* < 0.001), with reductions of 41.7% and 54.4% observed at 50 mg/kg, respectively (**Figures 3A, C**, and **Supplementary Table S1**). Cherry radish showed no significant changes in petiole number (*p* > 0.05; **Figure 3A**), but a 20.4% reduction in fruit diameter was observed at 100 mg/kg concentration (*p* > 0.05; **Figure 3C**; **Supplementary Tables S2**). Root length was inhibited in both species, with the largest reduction (14.2%) observed in Chinese cabbage at 10 mg/kg and a 35.0% reduction in cherry radish at 50 mg/kg (*p* > 0.05; **Figure 3B**; **Supplementary Tables S1, S2**). PS-MPs also significantly reduced leaf area in both crops (*p* < 0.05; **Figure 3D**). Chinese cabbage showed the greatest reduction (35.9%) at 10 mg/kg, while cherry radish exhibited the most significant decrease in leaf area at 50 mg/kg, with a reduction of 27.7% (**Supplementary Tables S1, S2**). The adverse morphological effects observed in cherry radish are likely attributable to the adsorption of PS-MPs onto root surfaces. Microscopic analysis confirmed MP accumulation on root surfaces under the 50 mg/kg and 100 mg/kg treatments (**Supplementary Figure S2**), consistent with previous studies conducted under hydrophobic conditions (Jiang et al., 2019; Li et al., 2023). Due to their relatively large size, micro-sized MPs tend to adhere externally to roots rather than penetrate internal tissues or translocate to above-ground parts (Chen et al., 2025). This surface adsorption can interfere with root physiological functions by inducing excessive production of reactive oxygen species (ROS) (Jia et al., 2023). Elevated ROS levels disrupt cell membrane integrity, damage proteins and DNA, and inhibit photosynthesis (Arshad et al., 2025). In addition, MP adsorption may physically block root capillaries, impairing water and nutrient uptake (Masciarelli et al., 2024). These combined physiological and structural disturbances likely account for the observed reductions in root length, fruit diameter, and leaf area of cherry radish under medium and high PS-MP concentrations. In contrast, Chinese cabbage exhibited minimal fluorescence signal from PS-MPs in root tissues, with only trace adsorption detected on the primary root surface at the highest concentration (**Supplementary Figure S3**). This suggests that MP adsorption is not the primary driver of morphological changes in Chinese cabbage. Notably, the negative effects in Chinese cabbage were more pronounced under low to medium PS-MP concentrations. We hypothesize that these effects are linked to alterations in soil structure. Specifically, the addition of PS-MPs reduced macroaggregate but increased microaggregate formation in soils planted with Chinese cabbage (**Supplementary Figure S4**). Macroaggregates typically harbor higher organic matter content, and their reduction is associated with decreased microbial biomass, diminished soil fertility, and consequently inhibited plant growth (Chen et al., 2024; Zhang and Liu, 2018).

Overall, while PS-MPs enhanced early seed germination by altering soil properties, they negatively affected plant morphology during later growth stages in our study. However, some previous studies have reported growth–promoting effects of PS–MPs, and the differences from our findings are likely attributable to variations in plant species and exposure concentrations (Zantis et al., 2023; Oladele et al., 2023). These contrasting findings highlight the challenge of balancing the observed beneficial and harmful impacts of MPs in agricultural systems, which is an important consideration for sustainable crop production. In addition, we observed a non-linear dose–response relationship, where low to medium concentrations of PS-MPs caused more pronounced morphological changes than higher concentrations in certain crops, emphasizing the potential risks associated with environmentally relevant exposure levels. To rigorously verify this non-linear pattern, future studies can apply quadratic or spline model fits to provide quantitative support and should focus on experimental conditions to better mimic real-world agricultural scenarios.

### 3.2 Physiological responses to PS-MP exposure in Chinese cabbage and cherry radish

**Figure 4:**
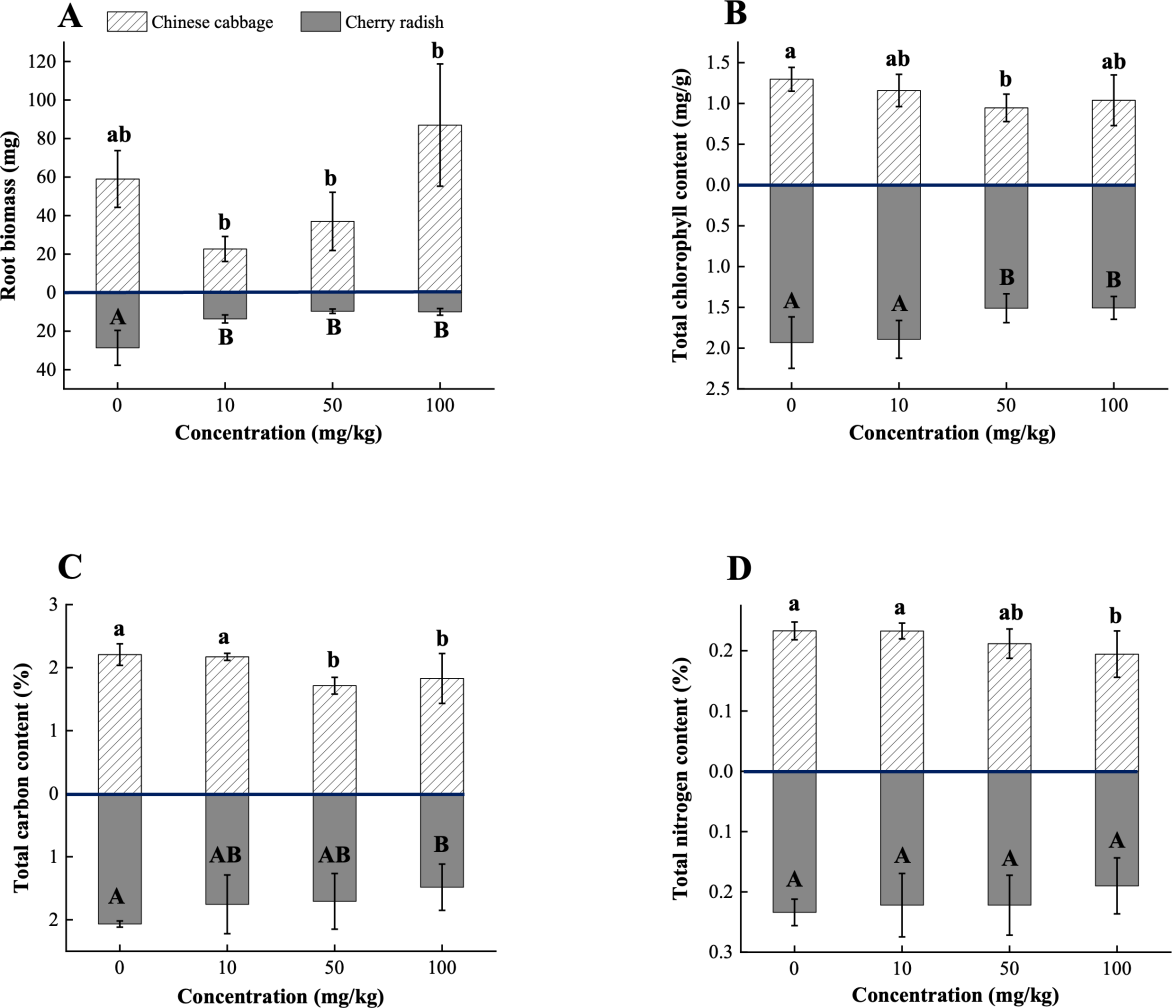
Physiological traits and soil properties of two edible plants under PS-MP treatment. **(A)** Root biomass; **(B)** Total chlorophyll content; **(C)** Total carbon content; **(D)** Total nitrogen content. [1] Results are presented as mean ± SD (Standard Deviation), N= 5. [2] Letter labels (e.g., a, b, ab/A, B, AB) are assigned using Duncan's post hoc test to indicate significant group differences. Groups sharing the same letter are not significantly different, while groups with different letters exhibit significant differences. [3] Statistical analyses are performed separately for Chinese cabbage and cherry radish, so uppercase and lowercase letter groupings represent independent tests for each species.

In Chinese cabbage, exposure to 10 mg/kg PS-MPs resulted in a 61.6% reduction in root biomass, while 100 mg/kg PS-MPs led to a 47.5% increase (*p* < 0.05; **Figure 4A**; **Supplementary Table S3**). This finding further emphasizes that the effects of MPs on plants may not follow a simple linear relationship. One possible explanation is that 10 mg/kg PS-MPs disrupted the soil macroaggregate structure (**Supplementary Figure S4**), which in turn may have altered the microbial community, reduced soil fertility, and inhibited root system development (En-Nejmy et al., 2024; Han et al., 2024). Conversely, at high concentrations, PS-MPs may enhance soil WHC, providing a more favorable environment for root growth, and thus contributing to the observed increase in root biomass (**Figure 2D**). Microscopic analysis further revealed the presence of PS-MPs (100 mg/kg) within the rhizosphere soil of Chinese cabbage (**Supplementary Figure S5**). These observations support the hypothesis that PS-MPs modulate soil moisture retention and create localized microenvironments conducive to plant growth (He et al., 2024). In contrast, cherry radish showed significant reductions in root biomass at all PS-MP concentrations (*p* < 0.05; **Figure 4A**; **Supplementary Table S4**). The decrease in root biomass is likely attributed to the mechanical blockage of root surfaces by PS-MPs, which interferes with the normal uptake of water and nutrients (**Supplementary Figure S2**). Additionally, the larger root surface area of cherry radish compared to Chinese cabbage makes it more susceptible to the adsorption of PS-MPs and other potential contaminants, such as pesticide residues and heavy metals (Jiang et al., 2024). The accumulation of these pollutants on root surfaces may exert more pronounced toxic effects, further contributing to the observed reductions in its root biomass.

**Figure 5:**
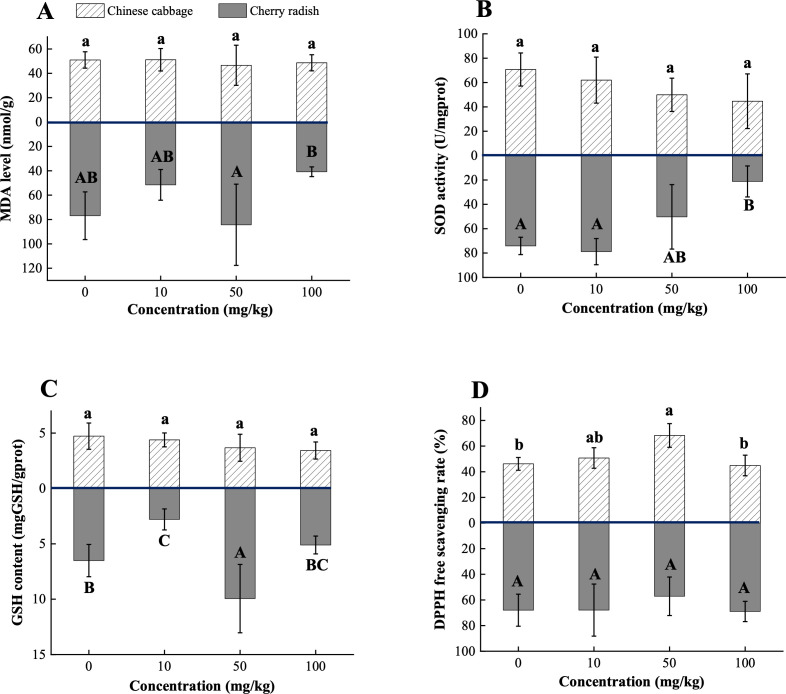
Biochemical traits of two edible plants under PS-MP treatments. **(A)** MDA level; **(B)** SOD activity; **(C)** GSH content; **(D)** DPPH free scavenging rate. [1] Results are presented as mean ± SD (Standard Deviation), N= 5. [2] Letter labels (e.g., a, b, ab/A, B, AB) are assigned using Duncan's post hoc test to indicate significant group differences. Groups sharing the same letter are not significantly different, while groups with different letters exhibit significant differences. [3] Statistical analyses are performed separately for Chinese cabbage and cherry radish, so uppercase and lowercase letter groupings represent independent tests for each species.

Exposure to PS-MPs significantly reduced total chlorophyll content in both Chinese cabbage and cherry radish (**Figure 4B**), with the maximum reduction observed at the medium concentration. Specifically, Chinese cabbage showed a 26.9% decrease, while cherry radish exhibited a 21.8% decrease (*p* < 0.05; **Supplementary Tables S3, S4**). These reductions can be attributed to the following factors: (1) Morphological changes. PS-MP exposure significantly decreased leaf area in both Chinese cabbage and cherry radish (*p* < 0.05; **Figure 3D**). Reduced leaf area directly limits the plant’s capacity to capture light energy, thereby lowering photosynthetic efficiency (Kang et al., 2023). Additionally, smaller leaf area is likely associated with a reduced number of stomata, which could further restrict carbon dioxide uptake, an essential substrate for photosynthesis (Haworth et al., 2023). This dual limitation on light capture and carbon dioxide assimilation finally inhibits chlorophyll synthesis. (2) Soil nutrient deficiencies. PS-MP exposure caused a significant reduction in TC and TN content in the planting soil, suggesting that MPs may potentially disrupt nutrient cycling by affecting soil microbial communities or organic matter decomposition (*p* < 0.05; **Figures 4C, D**). These findings are consistent with Saha et al. (2024), who reported that 5% w/w and 10% w/w MP treatments significantly reduced available N levels, leading to decreases in leaf area and chlorophyll content in mustard (*Brassica juncea*) and tomato (*Lycopersicum solanaceae*). C and N are essential elements for plant photosynthesis and metabolism. A reduction in C may limit the availability of C skeleton molecules needed for metabolic processes, while decreased N levels directly impair chlorophyll biosynthesis, as N is a core component of the chlorophyll molecule (Bassi et al., 2018). Additionally, diminished soil nutrient levels may weaken root absorption capabilities, compounding the negative impacts on plant growth and photosynthetic function (Bhat et al., 2024). (3) Oxidative stress and physical blockage. At 50 mg/kg PS-MPs, Chinese cabbage and cherry radish exhibited distinct oxidative stress responses, highlighting species-specific physiological regulation mechanisms. In Chinese cabbage, the significantly increased DPPH free radical scavenging activity suggests an activation of the antioxidant system to mitigate ROS accumulation (*p* < 0.05; **Figure 5D**). However, this stress response often requires the diversion of internal resources, potentially hindering chlorophyll synthesis. Conversely, cherry radish displayed elevated MDA levels at the same concentration, indicating oxidative damage to membrane lipids (*p* > 0.05; **Figure 5A**). Such damage may compromise the structural stability and functionality of chlorophyll molecules (Yang et al., 2021). In addition to oxidative stress, MP accumulation on root surfaces may compromise water and nutrient transport between the plant and rhizosphere. As reported by Ceccanti et al. (2024), such physical obstruction can reduce the uptake of Mg, which is the essential factor for plants in chlorophyll biosynthesis.

The results reinforce that plant physiological responses to PS–MPs are nonlinear and vary among species. Consistent with our findings, Zantis et al. (2023) observed relatively minor effects of PS-MPs on monocotyledonous plants (e.g., wheat and barley) but more pronounced responses in dicotyledonous species (e.g., lettuce and carrot). These findings emphasize the need to account for plant diversity when evaluating the ecological risks of MPs in agricultural ecosystems. Furthermore, they deepen our understanding of soil–plant–MP interactions and offer valuable insights into the underlying mechanisms.

### 3.3 Oxidative stress and biochemical responses induced by PS-MPs in Chinese cabbage and cherry radish

Under treatment with 50 mg/kg PS-MPs, the MDA level in cherry radish increased (*p* > 0.05; **Figure 5A**). This is likely attributable to the accumulation of PS-MPs on the root surface, which induces excessive production of ROS. The elevated ROS disrupts the lipid bilayer structure of cell membranes, triggering lipid peroxidation. Notably, 50 mg/kg PS-MPs also significantly stimulated the accumulation of GSH in cherry radish, suggesting that plants deploy non-enzymatic antioxidant mechanisms to dynamically respond to oxidative stress (*p* < 0.05; **Figure 5C**). This response may involve the reduction of hydrogen peroxide (H_2_O_2_) by GSH and the repair of damaged protein sulfhydryl groups, thus safeguarding cell membrane integrity (Jomova et al., 2023). However, at 100 mg/kg PS-MPs, MDA levels did not increase further; instead, they declined by 47.0% relative to the control (*p* > 0.05; **Figure 5A**; **Supplementary Table S6**). This observation may reflect broader metabolic disruptions induced by high concentrations of PS-MPs, which likely weaken non-oxidative stress pathways, such as water and nutrient uptake (Chang et al., 2024). In such conditions, plants may prioritize essential metabolic processes by downregulating growth, resulting in no further MDA accumulation. This hypothesis is supported by previous observations, which indicate a pronounced inhibitory effect of high PS-MPs concentrations on the morphology in cherry radish (**Figure 3**).

In contrast, MDA levels in Chinese cabbage showed no significant changes under PS-MPs exposure, consistent with microscopic observations that revealed no substantial accumulation of PS-MPs on its root surfaces (*p* > 0.05; **Figure 5A**; **Supplementary Figure S3**). This absence of significant lipid peroxidation may also align with the general understanding that micron-sized PS-MPs, due to their larger size, are less likely to penetrate plant tissues through apoplastic or symplastic pathways, and their toxicity is generally lower than that of nanoparticles (Li et al., 2020a; Rong et al., 2024). However, both SOD activity and GSH content in Chinese cabbage exhibited a declining trend with increasing PS-MPs concentrations, decreasing by 36.8% and 27.5%, respectively, at 100 mg/kg (*p* > 0.05; **Figures 5B, C**; **Supplementary Table S5**). This pattern suggests that low concentrations of PS-MPs may initially activate antioxidant systems, including SOD and GSH, to mitigate ROS and maintain cellular redox balance. However, as PS-MPs concentrations increase, antioxidant systems may become progressively depleted, leading to diminished SOD activity and GSH content. High PS-MPs concentrations may also alter the ROS profile; for example, reduced superoxide anion (O_2_^−^) levels alongside increased H_2_O_2_ levels could reduce the functional demand for SOD (Juan et al., 2021). Concurrently, the decline in GSH levels may reflect its extensive utilization in scavenging oxidative by-products such as MDA.

Interestingly, under 50 mg/kg PS-MPs exposure, the DPPH radical scavenging rate in Chinese cabbage increased significantly by 48.0% (*p* < 0.01; **Figure 5D**; **Supplementary Table S5**). This response may indicate an adaptive strategy involving non-enzymatic antioxidant pathways, such as direct scavenging of free radicals by antioxidant compounds, to counter moderate oxidative stress while maintaining ROS homeostasis. Furthermore, this phenomenon could be linked to the plant’s specific regulatory responses to medium PS-MPs concentrations. Moderate ROS levels, induced by PS-MPs exposure, may not overwhelm the antioxidant system but instead trigger signaling pathways that enhance antioxidant capacity. For instance, activation of the NADPH oxidase pathway may enable ROS to function as signaling molecules, regulating the expression of antioxidant-related genes and consequently improving the DPPH scavenging ability (Rohman et al., 2024).

Biochemical responses serve as a central regulatory mechanism in plants’ adaptation to PS-MPs stress, closely linked to morphological and physiological changes. These responses not only reveal the plants’ capacity to regulate oxidative stress but also highlight variations in adaptive strategies under different stress intensities. The interplay between antioxidant enzymes, non-enzymatic antioxidants, and free radical scavenging exhibits nonlinear characteristics, potentially involving shifts in resource allocation priorities and rebalancing of stress signaling pathways. Integrating biochemical responses with morphological and physiological traits can provide a comprehensive understanding of plant adaptation mechanisms under PS-MPs stress and offers novel insights into the intrinsic dynamics of plant-MP interactions. However, the present results only represent the endpoint level of biochemical responses and therefore do not capture the temporal dynamics of oxidative stress. Future studies incorporating time-course experiments are recommended to elucidate how antioxidant activity and oxidative damage evolve across different plant species under MP exposure.

### 3.4 Linking ecotoxicological mechanisms to species-specific plant responses

HRA supported the existence of species-specific ecotoxicological pathways in response to PS-MP stress (**Table 1**; **Supplementary Figures S6, S7**). In Chinese cabbage, key morphological (e.g., number of petioles) and physiological (e.g., Chlb content) traits showed △R² values above 0.50, indicating a strong influence from biochemical stress markers. Although PS-MPs were not visibly accumulated on root surfaces, oxidative stress appeared to play a central role. Disruptions in soil structure and nutrient availability may have altered root-zone conditions, leading to elevated ROS levels and the depletion of antioxidant defenses. This was further supported by chlorophyll reductions potentially caused by oxidative damage to chloroplast membranes and biosynthetic enzymes.

**Table 1:** Hierarchical regression analysis (HRA) revealing species-specific ecotoxicological mechanisms in response to PS-MP exposure.

HRA results	Chinese cabbage			Cherry radish		
	R1^2^	R2^2^	▵R^2^	R1^2^	R2^2^	▵R^2^
Root length	0.01	0.25	0.24	0.29	0.39	0.10
Number of petioles	0.02	0.54	0.52	n/a	n/a	n/a
Stem/fruit diameter	0.18	0.58	0.40	0.19	0.49	0.30
Leaf area	0.10	0.33	0.23	0.13	0.48	0.35
Root biomass	0.31	0.74	0.43	0.43	0.68	0.25
Chla content	0.10	0.48	0.38	0.57	0.76	0.19
Chlb content	0.20	0.71	0.51	0.65	0.74	0.09
Total chlorophyll content	0.12	0.53	0.41	0.61	0.77	0.16
Chlorophyll ratio	0.32	0.52	0.30	0.61	0.69	0.08

[1] R_1_²: Coefficient of determination representing the effect of PS-MP concentration on plant growth.

[2] R_2_²: Coefficient of determination representing the combined effects of PS-MP concentration and biochemical regulatory on plant growth.

[3] ▵R²: Incremental coefficient of determination (R_2_² - R_1_²), reflecting the unique contribution of biochemical regulatory to plant growth, independent of PS-MP concentration effects. A ΔR² value greater than 0.50 suggests that biochemical regulation plays a dominant role in mediating plant responses to PS-MP stress.

[4] The number of petioles in cherry radish remained constant across different PS-MP concentration treatments, and thus R_1_², R_2_², and ▵R² are not applicable for this parameter.

In contrast, cherry radish exhibited △R² values below 0.50 across all endpoints, suggesting that biochemical regulation had minimal influence. Microscopic observations confirmed substantial PS-MP accumulation on root surfaces at medium and high concentrations. These particles likely caused direct physical blockage, impairing water and nutrient uptake. As a result, nutrient deficiencies such as Mg or N may limit chlorophyll synthesis and plant productivity. This highlights physical toxicity as the primary mechanism in cherry radish.

Overall, the HRA results reinforce the broader conclusion that Chinese cabbage relies more on internal biochemical adjustments to cope with stress, while cherry radish is more susceptible to mechanical interference from particle accumulation. These findings provide a mechanistic explanation for the observed interspecific variation in MP-induced phytotoxicity.

## 4 Conclusion

This study explores the significant impacts of MPs across concentration gradients on the morphology, physiological, and biochemical traits of two common edible plants. MPs were found to enhance seed early germination by altering soil properties such as pH and water-holding capacity. However, in later stages, MPs exert inhibitory effects on plant growth by directly adhering to root surfaces or indirectly modifying soil aggregate fractions or element stocks. Notably, under the soil conditions tested in this study, plant responses to MPs exhibited a nonlinear dose–response relationship, which differed from patterns often reported under hydroponic conditions. At medium concentrations, both Chinese cabbage and cherry radish displayed pronounced negative effects, including reduced leaf area, diminished chlorophyll content, and antioxidant system failure. These findings underscore the heightened risks posed by environmentally relevant MP levels compared to previously studied higher concentrations. HRA analysis further revealed species-specific mechanisms underlying plant responses to MP stress. Chinese cabbage primarily relied on biochemical pathways, such as activation of antioxidant systems, to mitigate MP-induced stress, which was primarily attributed to the indirect effects of MPs altering soil properties. In contrast, cherry radish exhibited stronger direct adsorption effects, consistent with microscopic evidence of MP accumulation on root surfaces at medium to high concentrations. This mechanical blockage not only induced toxic effects but also obstructed nutrient dynamics, leading to impaired performance. These findings suggest that future research should prioritize environmentally relevant MP concentrations to better assess their impacts on agricultural ecosystems and clarify how species−specific traits influence the ecological toxicity of MPs. Such knowledge is essential for advancing sustainable agricultural practices and can guide the development of management strategies and policy decisions aimed at reducing plastic use in farming systems. Ultimately, these efforts can help balance crop productivity with the long−term preservation of soil health and environmental sustainability.

## Data Availability

The original contributions presented in the study are included in the article/**Supplementary Material**. Further inquiries can be directed to the corresponding author.

## References

[B1] Arshad M. Ma Y. Gao W. Zhang S. Shoaib M. Liu X.. (2025). Polypropylene microplastic exposure modulates multiple metabolic pathways in tobacco leaves, impacting lignin biosynthesis. Ecotoxicol. Environ. Saf. 292, 118005. doi: 10.1016/j.ecoenv.2025.118005, PMID: 40043503

[B2] Bassi D. Menossi M. Mattiello L. (2018). Nitrogen supply influences photosynthesis establishment along the sugarcane leaf. Sci. Rep. 8, 2327–2313. doi: 10.1038/s41598-018-20653-1, PMID: 29396510 PMC5797232

[B3] Bhat M. A. Mishra A. K. Shah S. N. Bhat M. A. Jan S. Rahman S.. (2024). Soil and mineral nutrients in plant health: A prospective study of iron and phosphorus in the growth and development of plants. Curr. Issues Mol. Biol. 46, 5194–5222. doi: 10.3390/cimb46060312, PMID: 38920984 PMC11201952

[B4] Boots B. Russell C. W. Green D. S. (2019). Effects of microplastics in soil ecosystems: above and below ground. Environ. Sci. Technol. 53, 11496–11506. doi: 10.1021/acs.est.9b03304, PMID: 31509704

[B5] Büks F. Kaupenjohann M. (2020). Global concentrations of microplastics in soils – a review. Soil 6, 649–662. doi: 10.5194/soil-6-649-2020

[B6] Ceccanti C. Davini A. Lo Piccolo E. Lauria G. Rossi V. Ruffini Castiglione M.. (2024). Polyethylene microplastics alter root functionality and affect strawberry plant physiology and fruit quality traits. J. Hazardous Mater. 470, 134164. doi: 10.1016/j.jhazmat.2024.134164, PMID: 38583200

[B7] Chang N. Chen L. Wang N. Cui Q. Qiu T. Zhao S.. (2024). Unveiling the impacts of microplastic pollution on soil health: A comprehensive review. Sci. Total Environ. 951, 175643. doi: 10.1016/j.scitotenv.2024.175643, PMID: 39173746

[B8] Chen Z. Carter L. J. Banwart S. A. Kay P. (2025). Microplastics in soil–plant systems: current knowledge, research gaps, and future directions for agricultural sustainability. Agron. (Basel) 15, 1519. doi: 10.3390/agronomy15071519

[B9] Chen Z. Carter L. J. Banwart S. A. Pramanik D. D. Kay P. (2024). Multifaceted effects of microplastics on soil-plant systems: Exploring the role of particle type and plant species. Sci. Total Environ. 954, 176641. doi: 10.1016/j.scitotenv.2024.176641, PMID: 39357762

[B10] De Silva Y. S. K. Rajagopalan U. M. Kadono H. Li D. (2022). Effects of microplastics on lentil (Lens culinaris) seed germination and seedling growth. Chemosphere (Oxford) 303, 135162. doi: 10.1016/j.chemosphere.2022.135162, PMID: 35654234

[B11] de Souza MaChado A. A. Lau C. W. Kloas W. Bergmann J. Bachelier J. B. Faltin E.. (2019). Microplastics can change soil properties and affect plant performance. Environ. Sci. Technol. 53, 6044–6052. doi: 10.1021/acs.est.9b01339, PMID: 31021077

[B12] En-Nejmy K. Hayany E. L. Al-Alawi M. Jemo M. Hafidi M. El Fels L. (2024). Microplastics in soil: A comprehensive review of occurrence, sources, fate, analytical techniques and potential impacts. Ecotoxicol. Environ. Saf. 288, 117332. doi: 10.1016/j.ecoenv.2024.117332, PMID: 39616787

[B13] Follmer C. M. Hummes A. P. Lângaro N. C. Petry C. Moterle D. F. Bortoluzzi E. C. (2021). Nutrient availability and pH level affect germination traits and seedling development of Conyza canadensis. Sci. Rep. 11, 15607–15614. doi: 10.1038/s41598-021-95164-7, PMID: 34341452 PMC8329304

[B14] Han L. Chen L. Feng Y. Kuzyakov Y. Chen Q. A. Zhang S.. (2024). Microplastics alter soil structure and microbial community composition. Environ. Int. 185, 108508. doi: 10.1016/j.envint.2024.108508, PMID: 38377723

[B15] Haworth M. Marino G. Materassi A. Raschi A. Scutt C. P. Centritto M. (2023). The functional significance of the stomatal size to density relationship: Interaction with atmospheric [CO2] and role in plant physiological behaviour. Sci. Total Environ. 863, 160908–160908. doi: 10.1016/j.scitotenv.2022.160908, PMID: 36535478

[B16] He M. Yao W. Meng Z. Liu J. Yan W. Meng W. (2024). Microplastic-contamination can reshape plant community by affecting soil properties. Ecotoxicol. Environ. Saf. 283, 116844. doi: 10.1016/j.ecoenv.2024.116844, PMID: 39128455

[B17] Horton A. A. Walton A. Spurgeon D. J. Lahive E. Svendsen C. (2017). Microplastics in freshwater and terrestrial environments: Evaluating the current understanding to identify the knowledge gaps and future research priorities. Sci. Total Environ. 586, 127–141. doi: 10.1016/j.scitotenv.2017.01.190, PMID: 28169032

[B18] Jia L. Liu L. Zhang Y. Fu W. Liu X. Wang Q.. (2023). Microplastic stress in plants: effects on plant growth and their remediations. Front. Plant Sci. 14. doi: 10.3389/fpls.2023.1226484, PMID: 37636098 PMC10452891

[B19] Jiang X. Chen H. Liao Y. Ye Z. Li M. Klobučar G. (2019). Ecotoxicity and genotoxicity of polystyrene microplastics on higher plant Vicia faba. Environ. pollut. (1987) 250, 831–838. doi: 10.1016/j.envpol.2019.04.055, PMID: 31051394

[B20] Jiang M. Zhao W. Liang Q. Cai M. Fan X. Hu S.. (2024). Polystyrene microplastics enhanced the toxicity of cadmium to rice seedlings: Evidence from rice growth, physiology, and element metabolism. Sci. Total Environ. 945, 173931. doi: 10.1016/j.scitotenv.2024.173931, PMID: 38885718

[B21] Jin T. Tang J. Lyu H. Wang L. Gillmore A. B. Schaeffer S. M. (2022). Activities of microplastics (MPs) in agricultural soil: A review of MPs pollution from the perspective of agricultural ecosystems. J. Agric. Food Chem. 70, 4182–4201. doi: 10.1021/acs.jafc.1c07849, PMID: 35380817

[B22] Jomova K. Raptova R. Alomar S. Y. Alwasel S. H. Nepovimova E. Kuca K.. (2023). Reactive oxygen species, toxicity, oxidative stress, and antioxidants: chronic diseases and aging. Arch. Toxicol. 97, 2499–2574. doi: 10.1007/s00204-023-03562-9, PMID: 37597078 PMC10475008

[B23] Juan C. A. Pérez de la Lastra J. M. Plou F. J. Pérez-Lebeña E. (2021). The chemistry of reactive oxygen species (ROS) revisited: outlining their role in biological macromolecules (DNA, lipids and proteins) and induced pathologies. Int. J. Mol. Sci. 22, 4642. doi: 10.3390/ijms22094642, PMID: 33924958 PMC8125527

[B24] Kang J. Chu Y. Ma G. Zhang Y. Zhang X. Wang M.. (2023). Physiological mechanisms underlying reduced photosynthesis in wheat leaves grown in the field under conditions of nitrogen and water deficiency. Crop J. 11, 638–650. doi: 10.1016/j.cj.2022.06.010

[B25] Khalid N. Aqeel M. Noman A. (2020). Microplastics could be a threat to plants in terrestrial systems directly or indirectly. Environ. pollut. (1987) 267, 115653. doi: 10.1016/j.envpol.2020.115653, PMID: 33254725

[B26] Li H. Chang X. Zhang J. Wang Y. Zhong R. Wang L.. (2023). Uptake and distribution of microplastics of different particle sizes in maize (Zea mays) seedling roots. Chemosphere (Oxford) 313, 137491. doi: 10.1016/j.chemosphere.2022.137491, PMID: 36493893

[B27] Li Z. Li Q. Li R. Zhao Y. Geng J. Wang G. (2020b). Physiological responses of lettuce (Lactuca sativa L.) to microplastic pollution. Environ. Sci. pollut. Res. Int. 27, 30306–30314. doi: 10.1007/s11356-020-09349-0, PMID: 32451901

[B28] Li L. Luo Y. Li R. Zhou Q. Peijnenburg W. J. G. M. Yin N.. (2020a). Effective uptake of submicrometre plastics by crop plants via a crack-entry mode. Nat. Sustainability 3, 929–937. doi: 10.1038/s41893-020-0567-9

[B29] Li W. Zhao J. Zhang Z. Ren Z. Li X. Zhang R.. (2024). Uptake and effect of carboxyl-modified polystyrene microplastics on cotton plants. J. Hazardous Mater. 466, 133581–133581. doi: 10.1016/j.jhazmat.2024.133581, PMID: 38271872

[B30] Lozano Y. M. Lehnert T. Linck L. T. Lehmann A. Rillig M. C. (2021). Microplastic shape, polymer type, and concentration affect soil properties and plant biomass. Front. Plant Sci. 12. doi: 10.3389/fpls.2021.616645, PMID: 33664758 PMC7920964

[B31] Lozano Y. M. Rillig M. C. (2020). Effects of microplastic fibers and drought on plant communities. Environ. Sci. Technol. 54, 6166–6173. doi: 10.1021/acs.est.0c01051, PMID: 32289223 PMC7241422

[B32] Masciarelli E. Casorri L. Di Luigi M. Beni C. Valentini M. Costantini E.. (2024). Microplastics in agricultural crops and their possible impact on farmers’ Health: A review. Int. J. Environ. Res. Public Health 22, 45. doi: 10.3390/ijerph22010045, PMID: 39857498 PMC11765068

[B33] Mathew J. Pulicharla R. Rezai P. Brar S. K. (2024). Microplastics in wastewaters: Pretreatment to detection trail. J. Water Process Eng. 64, 105702. doi: 10.1016/j.jwpe.2024.105702

[B34] Meizoso-Regueira T. Fuentes J. Cusworth S. J. Rillig M. C. (2024). Prediction of future microplastic accumulation in agricultural soils. Environ. pollut. (1987) 359, 124587. doi: 10.1016/j.envpol.2024.124587, PMID: 39038775

[B35] Meng F. Yang X. Riksen M. Xu M. Geissen V. (2021). Response of common bean (Phaseolus vulgaris L.) growth to soil contaminated with microplastics. Sci. Total Environ. 755, 142516. doi: 10.1016/j.scitotenv.2020.142516, PMID: 33045612

[B36] Oladele S. O. Ojokole W. Oladele B. B. (2023). Microplastics in agricultural soil: Polystyrene fragments inhibit soil microbial and enzymatic activities but promote nutrient concentration of Cowpea (Vigna unguiculata). J. Hazardous Mater. Adv. 10, 100263. doi: 10.1016/j.hazadv.2023.100263

[B37] Qi Y. Yang X. Pelaez A. M. Huerta Lwanga E. Beriot N. Gertsen H.. (2018). Macro- and micro- plastics in soil-plant system: Effects of plastic mulch film residues on wheat (Triticum aestivum) growth. Sci. Total Environ. 645, 1048–1056. doi: 10.1016/j.scitotenv.2018.07.229, PMID: 30248830

[B38] Qiang L. Hu H. Li G. Xu J. Cheng J. Wang J.. (2023). Plastic mulching, and occurrence, incorporation, degradation, and impacts of polyethylene microplastics in agroecosystems. Ecotoxicol. Environ. Saf. 263, 115274. doi: 10.1016/j.ecoenv.2023.115274, PMID: 37499389

[B39] Rico C. M. Majumdar S. Duarte-Gardea M. Peralta-Videa J. R. Gardea-Torresdey J. L. (2011). Interaction of nanoparticles with edible plants and their possible implications in the food chain. J. Agric. Food Chem. 59, 3485–3498. doi: 10.1021/jf104517j, PMID: 21405020 PMC3086136

[B40] Rillig M. C. (2020). Plastic and plants. Nature Sustainability 3 (11), 887–888. doi: 10.1038/s41893-020-0583-9

[B41] Rillig M. C. Lehmann A. (2020). Microplastic in terrestrial ecosystems. Sci. (American Assoc. Advancement Sci.) 368, 1430–1431. doi: 10.1126/science.abb5979, PMID: 32587009 PMC7115994

[B42] Rohman M. Islam M. Habib S. H. Choudhury D. A. Mohi-Ud-Din M. (2024). NADPH oxidase-mediated reactive oxygen species, antioxidant isozymes, and redox homeostasis regulate salt sensitivity in maize genotypes. Heliyon 10, e26920–e26920. doi: 10.1016/j.heliyon.2024.e26920, PMID: 38468963 PMC10926083

[B43] Rong S. Wang S. Liu H. Li Y. Huang J. Wang W.. (2024). Evidence for the transportation of aggregated microplastics in the symplast pathway of oilseed rape roots and their impact on plant growth. Sci. Total Environ. 912, 169419–169419. doi: 10.1016/j.scitotenv.2023.169419, PMID: 38128661

[B44] Saha A. Baruah P. Handique S. (2024). Assessment of microplastic pollution on soil health and crop responses: Insights from dose-dependent pot experiments. Appl. Soil Ecol. A. Section Agricult. Ecosyst. Environ. 203, 105648. doi: 10.1016/j.apsoil.2024.105648

[B45] Shorobi F. M. Vyavahare G. D. Seok Y. J. Park J. H. (2023). Effect of polypropylene microplastics on seed germination and nutrient uptake of tomato and cherry tomato plants. Chemosphere (Oxford) 329, 138679. doi: 10.1016/j.chemosphere.2023.138679, PMID: 37059201

[B46] Statista (2025). Annual production of plastics worldwide from 1950 to 2023. Available online at: https://www.statista.com/statistics/282732/global-production-of-plastics-since-1950/ (Accessed 20 March 2025).

[B47] Thompson R. C. Olsen Y. Mitchell R. P. Davis A. Rowland S. J. Anthony W. G.. (2004). Lost at sea: where is all the plastic? Sci. (American Assoc. Advancement Sci.) 304, 838–838. doi: 10.1126/science.1094559, PMID: 15131299

[B48] Wang J. Liu W. Zeb A. Wang Q. Mo F. Shi R.. (2024). Biodegradable microplastic-driven change in soil pH affects soybean rhizosphere microbial N transformation processes. J. Agric. Food Chem. 72, 16674–16686. doi: 10.1021/acs.jafc.4c04206, PMID: 39021203

[B49] Xiao W. Xiang P. Liao W. Xiong Z. Peng L. Zou L.. (2024). Effects of polystyrene microplastics on the growth and metabolism of highland barley seedlings based on LC-MS. Front. Plant Sci. 15. doi: 10.3389/fpls.2024.1477605, PMID: 39741681 PMC11685026

[B50] Yang X. Lu M. Wang Y. Wang Y. Liu Z. Chen S. (2021). Response mechanism of plants to drought stress. Horticulturae 7, 50. doi: 10.3390/horticulturae7030050

[B51] Zantis L. J. Rombach A. Adamczyk S. Velmala S. M. Adamczyk B. Vijver M. G.. (2023). Species-dependent responses of crop plants to polystyrene microplastics. Environ. pollut. 335, 122243. doi: 10.1016/j.envpol.2023.122243, PMID: 37482341

[B52] Zhang G. S. Liu Y. F. (2018). The distribution of microplastics in soil aggregate fractions in southwestern China. Sci. Total Environ. 642, 12–20. doi: 10.1016/j.scitotenv.2018.06.004, PMID: 29894871

[B53] Zhang Q. Zhao M. Meng F. Xiao Y. Dai W. Luan Y. (2021). Effect of polystyrene microplastics on rice seed germination and antioxidant enzyme activity. Toxics (Basel) 9, 179. doi: 10.3390/toxics9080179, PMID: 34437497 PMC8402430

