# Supplementary material for: Environmental levels of microplastics disrupt growth and stress pathways in edible crops via species-specific mechanisms
Source: Front Plant Sci. 2025 Aug 28;16:1670247. doi: 10.3389/fpls.2025.1670247 (PMC12424235; doi:10.3389/fpls.2025.1670247)
Supplement: Supplementary file 1 [file DataSheet1.pdf]

# *Supplementary Material*

## 1. Supplementary Texts

**Text S1.** Method for determining soil water-holding capacity (WHC).

20 g of air-dry soil (W) were placed in a funnel lined with filter paper. Then, 50 mL of deionized water (V1) was slowly poured into the funnel. Once the water had drained completely, the volume of water retained by the soil (V2) was recorded. The WHC was calculated using the following formula:

$WHC\ (\%) = [(V1 - V2)] / W \times 100$

Where: V1 is the initial volume of water added; V2 is the volume of water that drained, and W is the weight of the soil sample.

**Text S2.** Method for determining soil water-stable aggregates (WSA).

Soil samples were divided into five water stable aggregate fractions:

*i) Large macroaggregates (> 2000 μm)*

*ii) Medium macroaggregates (1000-2000 μm)*

*iii) Small macroaggregates (250-1000 μm)*

*iv) Microaggregates (53-250 μm)*

*v) Silt-clay-sized microaggregates and minerals (< 53 μm)*

A 2000 μm sieve was placed in a basin and filled with deionized water to submerge the sample completely. 40 g of air-dried soil were placed on the 2000 μm sieve and left to slake for 5 minutes, allowing the breakdown of unstable aggregates. Afterward, the soil was sieved manually by moving the sieve up and down 50 times over a period of 2 minutes. The remaining aggregates (> 2000 μm) were collected in a labeled bulk density tin (dimensions: 17.5 × 7 × 5.5 cm), after removing any floating organic material. This process was repeated with sieves of 1000 μm (40 strokes), 250 μm (30 strokes), and 53 μm (10 strokes), following the same procedure for each fraction. The aggregates in each size range (1000-2000 μm, 250-1000 μm, 53-250 μm, and < 53 μm) were collected in labeled bulk density tins. The aggregate fractions were then oven-dried separately at 60 °C for 24 hours, and the relevant data were recorded.

**Text S3.** ImageJ version information and protocol.

This file provides the ImageJ version information (version 1.54g) and a step-by-step image analysis protocol. Screenshots of parameter settings and a link to an instructional video are also included to facilitate reproducibility.

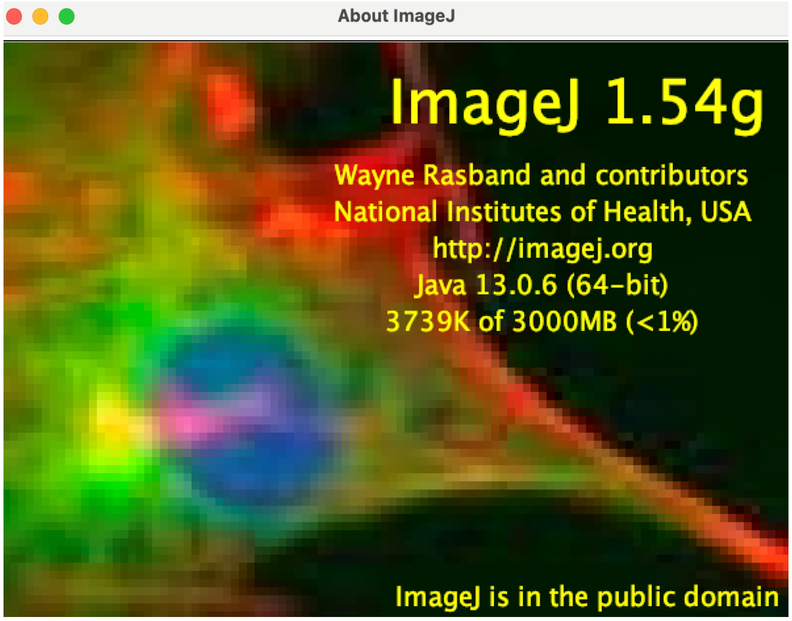


*Link 1: Root length, Image J, Arabidopsis, research, stress, analysis:*

https://www.youtube.com/watch?v=P3zC_bWBE8U

*Link 2: How to measure leaf area in ImageJ program:*

https://www.youtube.com/watch?v=kSSkOmQxdPM

**Text S4.** Equations for calculating Chla, Chlb, total chlorophyll content, and chlorophyll ratio.

*Chla (mg/g) = (11.93 × A664-1.93 × A647) × V/ (1000 × W)*

*Chlb (mg/g) = (20.36 × A647-5.5 × A664) × V/ (1000 × W)*

*Total Chlorophyll (mg/g) = Chla+Chlb*

*Chlorophyll ratio = Chla/Chlb*

Where: V is the volume of 90% acetone added; W is the fresh weight of plant leaves; A664 is the absorbance at 664 nm, and A647 is the absorbance at 647 nm.

**Text S5.** Method for determining MDA level.

The thiobarbituric acid (TBA) method was used to assess MDA content in plant leaves. A 0.5 g sample of leaves was weighed and soaked in 4.5 mL of MDA extraction buffer. The leaves were ground into a paste using a mortar and pestle, followed by centrifugation at 2500 rpm for 10 minutes. Working solutions and standard reagents were prepared according to the kit instructions. Subsequently, 50 μL of the supernatant was mixed with 1000 μL of the working solution in a centrifuge tube covered with aluminum foil (with a small hole for ventilation). The mixture was heated above 95°C for 20 minutes and then cooled. Absorbance at 530 nm was measured using a Jasco scanning spectrophotometer. MDA content was calculated according to the formula provided in the assay kit.

$$MDA\ content\ (nmol/g) = \frac{Abs\ (sample) - Abs\ (blank)}{Abs\ (standard) - Abs\ (blank)}\ \text{x}\ 10 \div \frac{0.5}{4.5}$$

**Text S6.** Method for determining SOD activity.

The xanthine oxidase method was employed to measure SOD activity in plant leaves. A 0.5 g sample was weighed and soaked in 4.5 mL of 90% saline solution. The leaves were ground into a paste using a mortar and pestle, and the mixture was centrifuged at 2500 rpm for 10 minutes. Working solutions and standard reagents were prepared according to the kit instructions. A 50 μL aliquot of the supernatant was combined with 1300 μL of the working solution and heated at 37°C for 40 minutes. Following the incubation, 2 mL of chromogen solution was added and mixed, and the solution was incubated at room temperature for 10 minutes. Absorbance at 550 nm was measured using a Jasco scanning spectrophotometer. SOD activity was calculated based on the formula provided in the assay kit.

$$SOD\ activity\ (U/mgprot) = \frac{Abs\ (control) - Abs\ (sample)}{Abs\ (control)} \div 50\%\ \text{x}\ \frac{3.35}{0.05} \div \text{Cpr}$$

**Text S7.** Method for determining GSH content.

The dithionitrobenzoic acid (DTNB) method was used to assess GSH content in plant leaves. A 0.5 g sample was weighed and soaked in 4.5 mL of 90% saline solution. After the leaves were ground into a paste using a mortar and pestle, the extract was centrifuged at 2500 rpm for 10 minutes. Working solutions and standard reagents were prepared as per the kit instructions. A 0.5 mL aliquot of the homogenate was mixed with 2 mL of reaction solution. Then, 1 mL of the supernatant was mixed with 1.55 mL of the working solution and allowed to sit at room temperature for 5 minutes. Absorbance at

420 nm was measured using a Jasco scanning spectrophotometer. GSH content was calculated using the following formula:

$$GSH\ content\ (mgGSH/gprot) = \frac{Abs\ (sample) - Abs\ (blank)}{Abs\ (standard) - Abs\ (blank)}\ x\ 20\ x\ 10^{-3}\ x\ 307\ x\ 5 \div Cpr$$

**Text S8.** Method for determining 2,2-diphenyl-1-picrylhydrazyl (DPPH) free radical scavenging rate. To determine DPPH free radical scavenging capacity, 0.2 g of plant leaves were weighed and soaked in 4 mL of 80% methanol. The leaves were ground into a paste using a mortar and pestle, and the mixture was centrifuged at 2500 rpm for 10 minutes. A 400 μL aliquot of the supernatant was mixed with 600 μL of DPPH solution, and the mixture was kept in the dark for 30 minutes. Absorbance at 517 nm was measured using a Jasco scanning spectrophotometer. The DPPH free radical scavenging rate was calculated using the formula provided in the assay kit.

$$DPPH\ free\ scavenging\ rate\ (\%) = (1 - (Abs_{sample} - Abs_{control}) \div Abs_{blank}) \times 100$$

## 2. Supplementary Figures

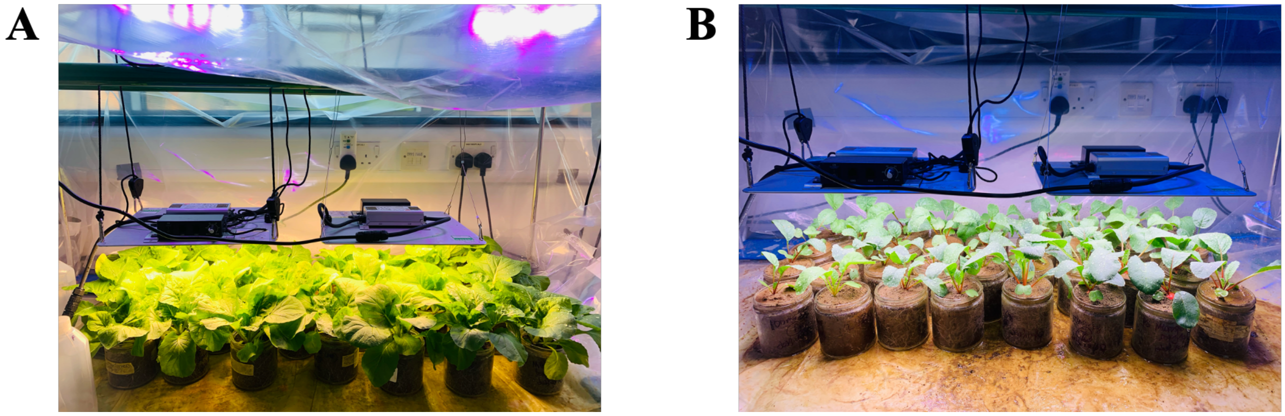

**Figure S1.** Two consecutive soil incubation experiments conducted in a greenhouse. (A) Chinese cabbage cultivation; (B) Cherry radish cultivation.

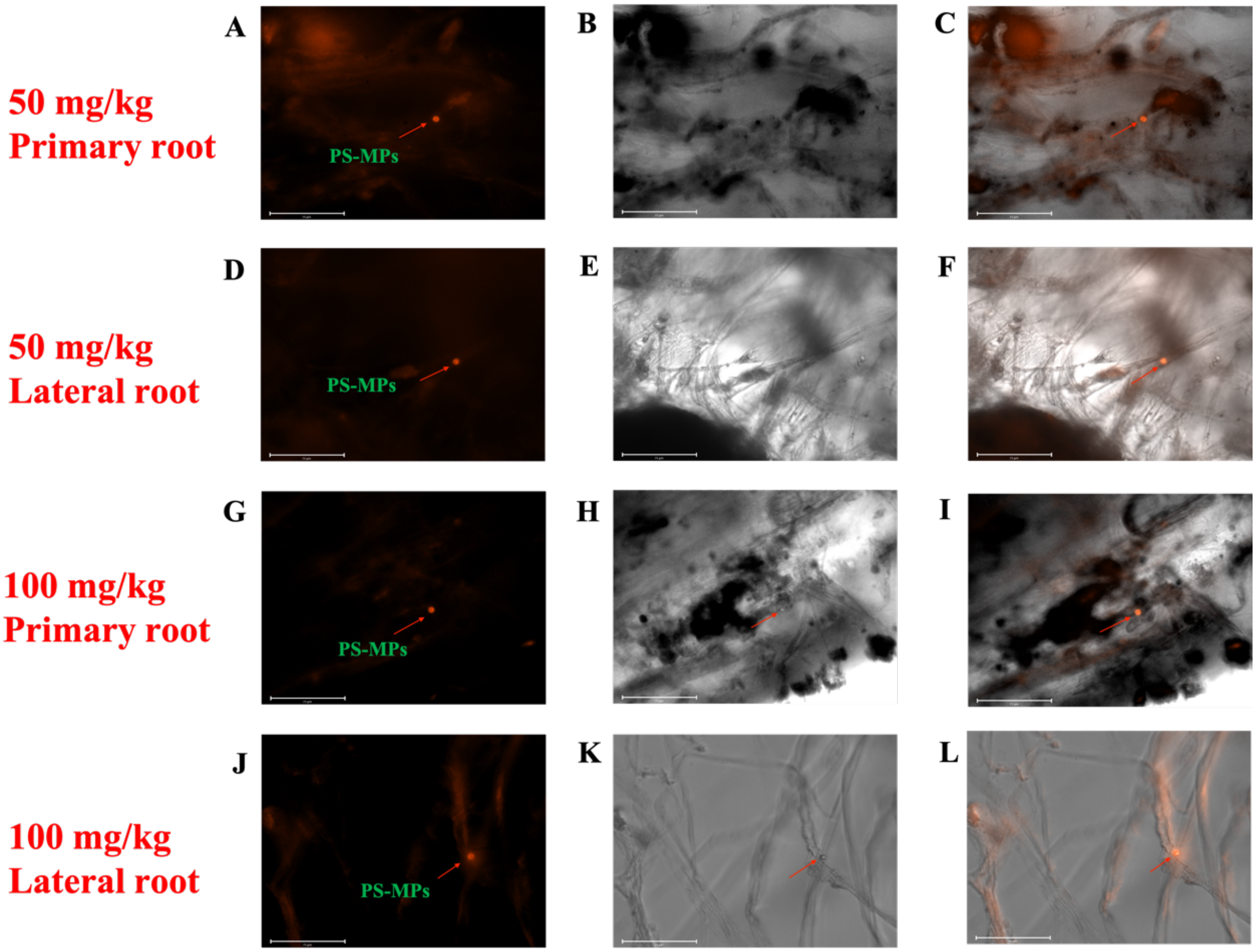


**Figure S2.** Detection of 5 µm fluorescent PS-MPs on the root surfaces of cherry radish using EVOS FL Auto 2 imaging. All images were captured at 40X magnification, 75 µm. Each line represents images taken from the same field of view. (A), (D), (G), and (J) were captured using the RFP light cube; (B), (E), (H), and (K) were captured using the Trans light cube; (C), (F), (I), and (L) represent images overlapped by the RFP and Trans light cubes.

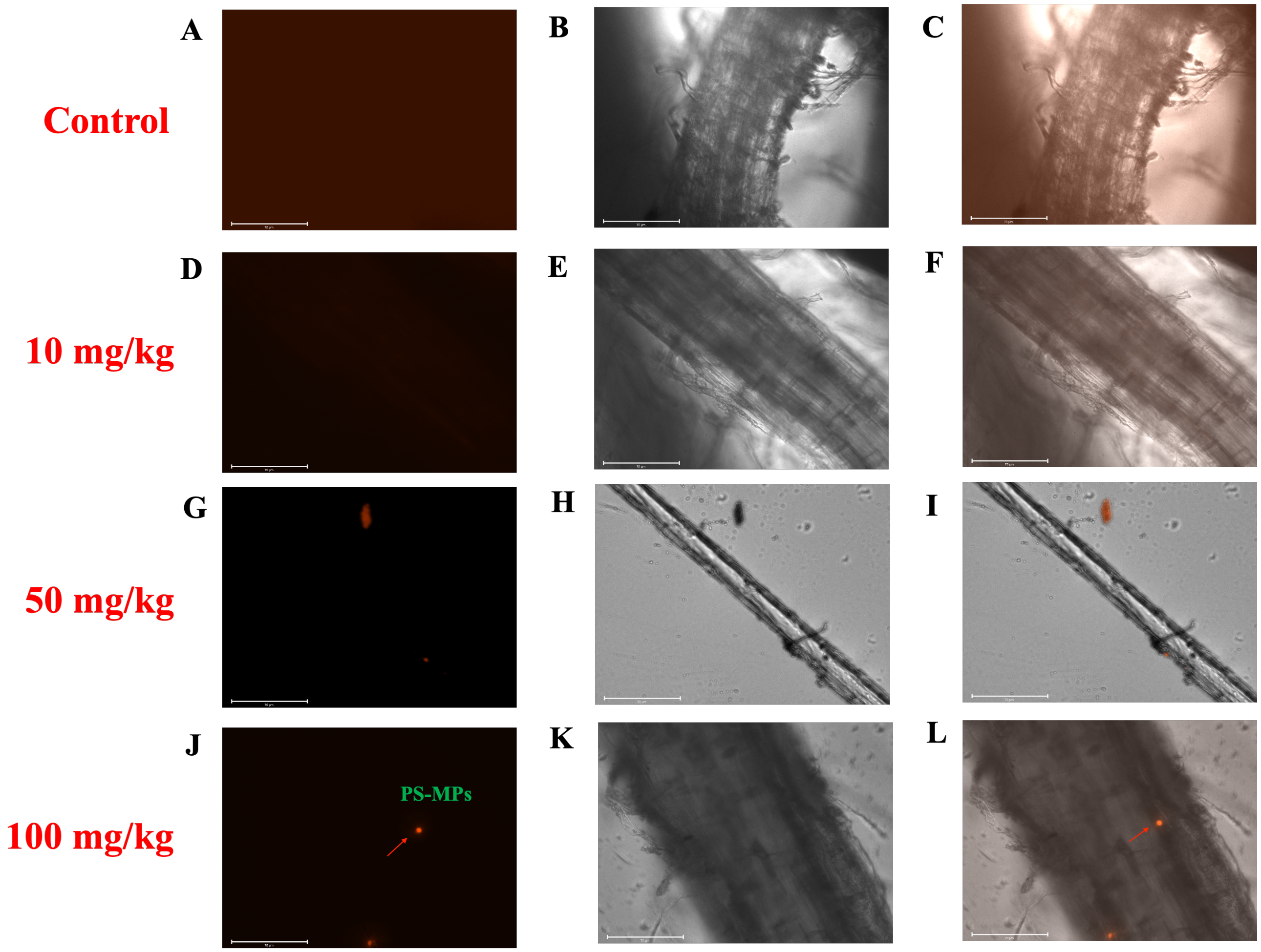


**Figure S3.** Detection of 5 μm fluorescent PS-MPs on the root surfaces of Chinese cabbage using EVOS FL Auto 2 imaging. All images were captured at 40X magnification, 75 μm. Each line represents images taken from the same field of view. (A), (D), (G), and (J) were captured using the RFP light cube; (B), (E), (H), and (K) were captured using the Trans light cube; (C), (F), (I), and (L) represent images overlapped by the RFP and Trans light cubes.

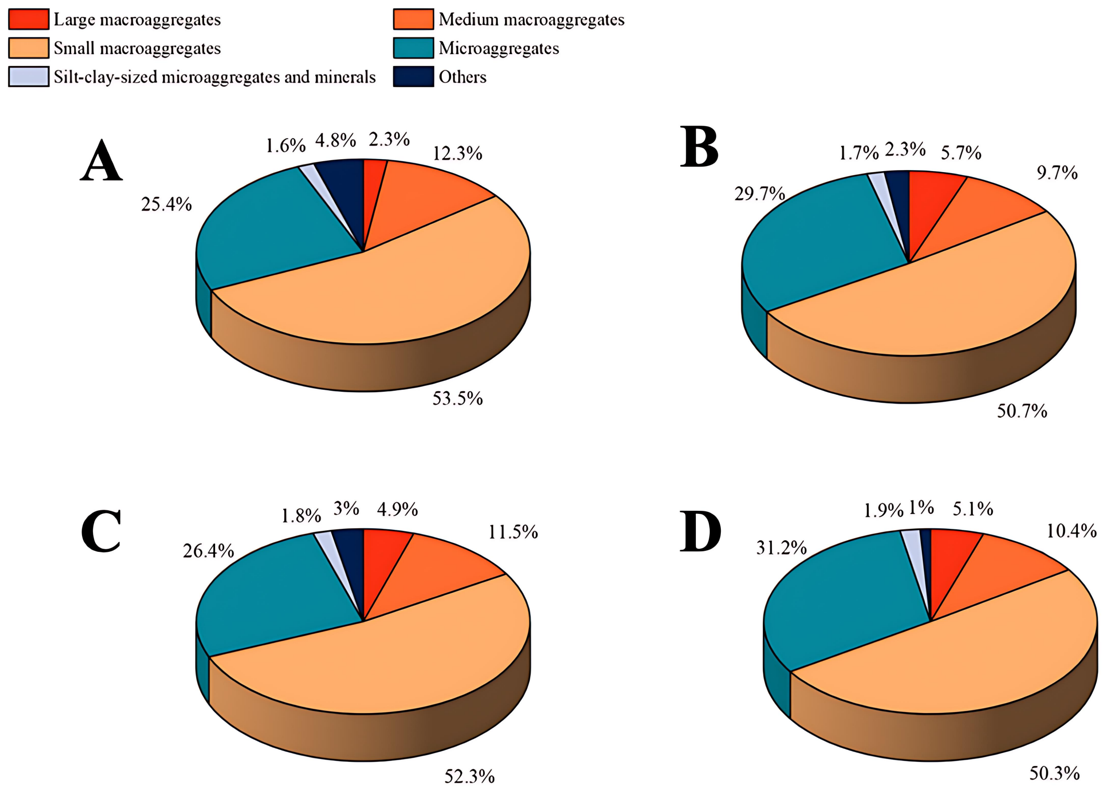


**Figure S4.** Water-table aggregates (WSA) fractions in Chinese cabbage planting soil under PS-MPs treatments. (A) Control treatment; (B) 10 mg/kg treatment; (C) 50 mg/kg treatment; (D) 100 mg/kg treatment.

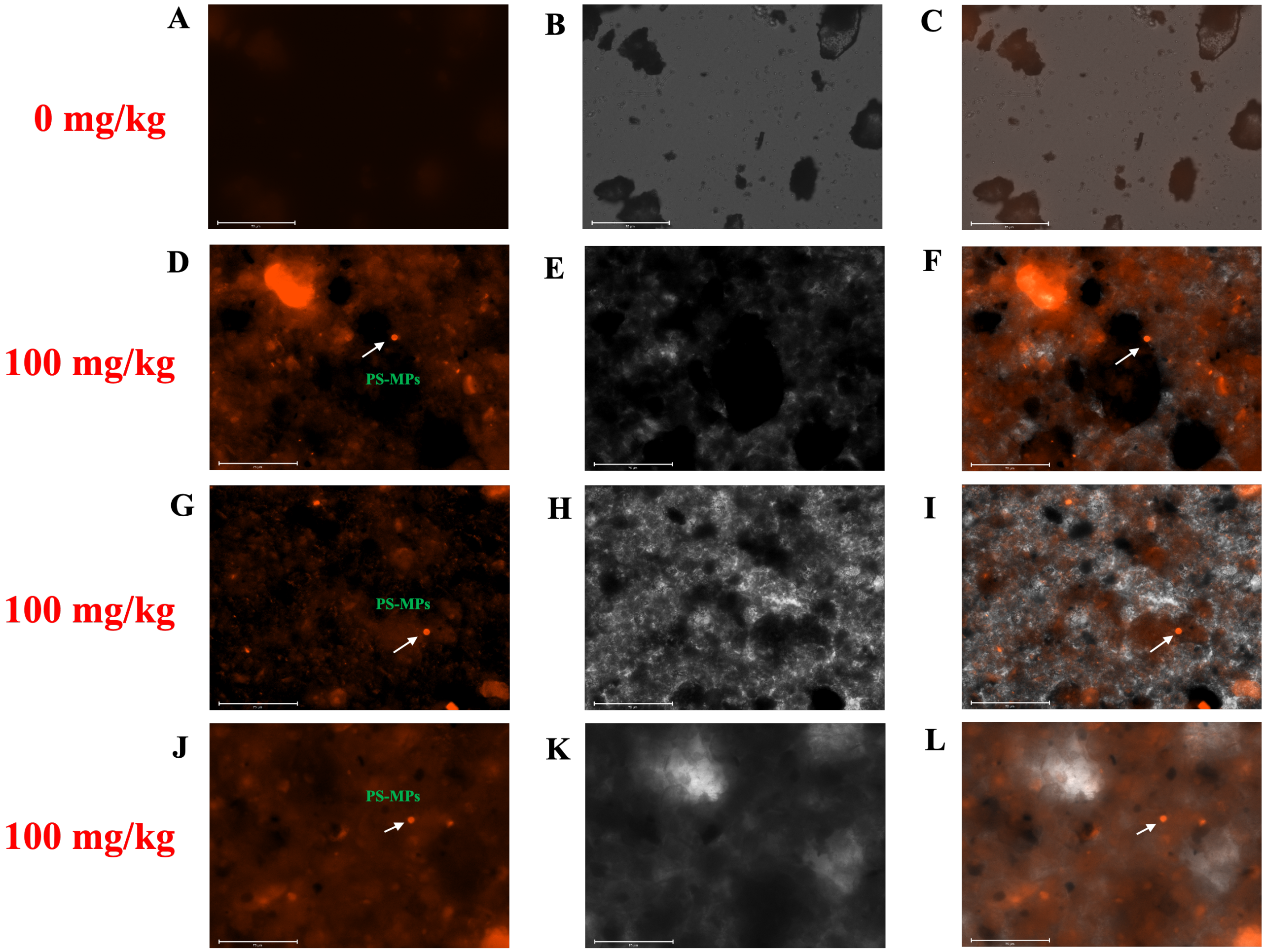


**Figure S5.** Detection of 5 μm fluorescent PS-MPs in Chinese cabbage rhizosphere soil using EVOS FL Auto 2 imaging. All images were captured at 40X magnification, 75 μm. Each line represents images taken from the same field of view. (A), (D), (G), and (J) were captured using the RFP light cube; (B), (E), (H), and (K) were captured using the Trans light cube; (C), (F), (I), and (L) represent images overlapped by the RFP and Trans light cubes.

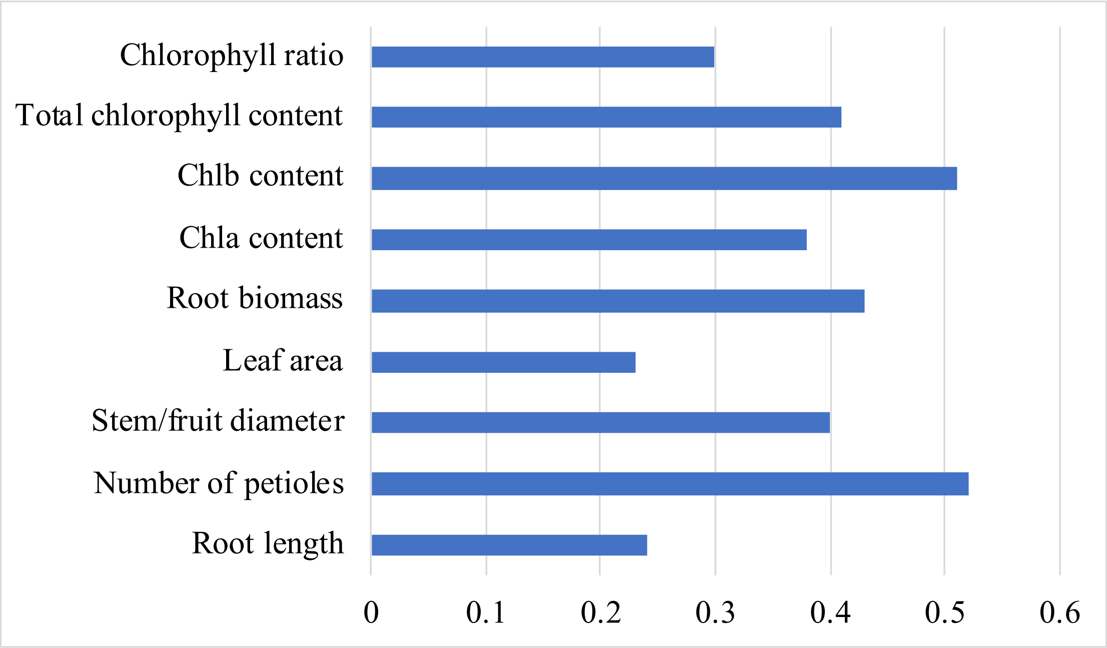


**Figure S6.** Bar chart of $\Delta R^2$ values from hierarchical regression analysis (HRA) for Chinese cabbage under PS-MP exposure. *$\Delta R^2$: Incremental coefficient of determination ($R_2^2$ - $R_1^2$), reflecting the unique contribution of biochemical regulatory to plant growth, independent of PS-MP concentration effects. A $\Delta R^2$ value greater than 0.50 suggests that biochemical regulation plays a dominant role in mediating plant responses to PS-MP stress.

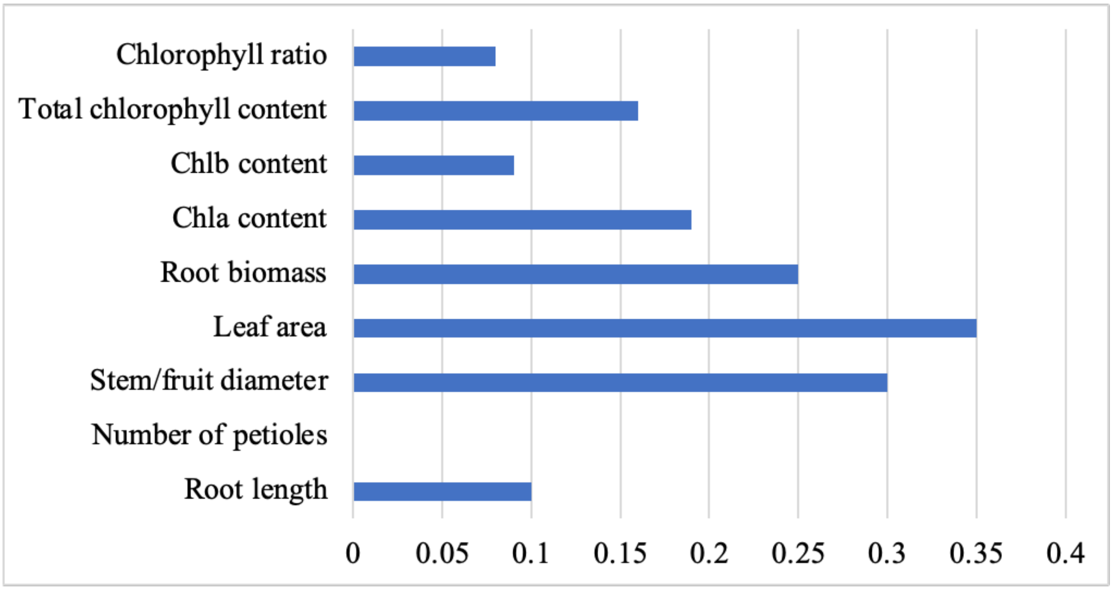


**Figure S7.** Bar chart of $\Delta R^2$ values from hierarchical regression analysis (HRA) for cherry radish under PS-MP exposure. *$\Delta R^2$: Incremental coefficient of determination ($R_2^2$ - $R_1^2$), reflecting the unique contribution of biochemical regulatory to plant growth, independent of PS-MP concentration effects. A $\Delta R^2$ value greater than 0.50 suggests that biochemical regulation plays a dominant role in mediating plant responses to PS-MP stress. * The number of petioles in cherry radish remained constant across different PS-MP concentration treatments, thus $\Delta R^2$ is not applicable for this parameter.

# 3. Supplementary Tables

**Table S1.** Morphological endpoints of Chinese cabbage under varying concentrations of PS-MPs.

| Treatment | Number of petioles | Root length (cm) | Stem diameter (cm) | Leaf area ($cm^2$) |
|---|---|---|---|---|
| Control | 7.20±0.84$^{a}$ | 7.04±0.29$^{a}$ | 0.68±0.10$^{a}$ | 27.89±5.21$^{a}$ |
| 10mg/kg | 5.20±0.84$^{bc}$ | 6.04±0.92$^{a}$ | 0.45±0.13$^{b}$ | 17.98±4.63$^{b}$ |
| 50mg/kg | 4.20±1.10$^{c}$ | 6.28±0.88$^{a}$ | 0.31±0.12$^{b}$ | 21.61±5.29$^{ab}$ |
| 100mg/kg | 5.80±1.39$^{b}$ | 6.07±.0.77$^{a}$ | 0.38±0.12$^{b}$ | 25.07±4.99$^{a}$ |
| *F* | 9.535 | 1.880 | 9.724 | 3.638 |
| *p* | 0.001 | 0.174 | 0.001 | 0.036 |

*[1] Results are presented as mean ± SD (Standard Deviation), N = 5.*

*[2] F-value: Indicates the ratio of between-group variance to within-group variance, assessing whether there are significant differences between groups.*

*[3] p-value: Reflects the statistical significance of the observed differences; a smaller p-value suggests significant differences.*

*[4] Letter labels (e.g., a, b, ab) were applied using Duncan's post hoc test to indicate specific group differences. Groups with the same letter are not significantly different, whereas those with different letters exhibit significant differences.*

**Table S2.** Morphological endpoints of cherry radish under varying concentrations of PS-MPs.

| Treatment | Number of petioles | Root length (cm) | Stem diameter (cm) | Leaf area ($cm^2$) |
|---|---|---|---|---|
| Control | 6±0 | $6.21 \pm 2.07^{A}$ | $2.75 \pm 0.58^{A}$ | $19.93 \pm 3.47^{A}$ |
| 10mg/kg | 6±0 | $5.13 \pm 1.31^{A}$ | $2.38 \pm 0.54^{A}$ | $15.14 \pm 2.04^{B}$ |
| 50mg/kg | 6±0 | $4.04 \pm 0.50^{A}$ | $2.72 \pm 0.19^{A}$ | $14.42 \pm 2.06^{B}$ |
| 100mg/kg | 6±0 | $5.34 \pm 2.41^{A}$ | $2.19 \pm 0.25^{A}$ | $15.05 \pm 2.75^{B}$ |
| *F* | n/a | 1.320 | 1.534 | 4.652 |
| *p* | n/a | 0.303 | 0.244 | 0.016 |

*[1] Results are presented as mean ± SD (Standard Deviation), N = 5.*

*[2] F-value: Indicates the ratio of between-group variance to within-group variance, assessing whether there are significant differences between groups.*

*[3] p-value: Reflects the statistical significance of the observed differences; a smaller p-value suggests significant differences.*

*[4] Letter labels (e.g., A, B, AB) were applied using Duncan's post hoc test to indicate specific group differences. Groups with the same letter are not significantly different, whereas those with different letters exhibit significant differences.*

*[5] The number of petioles in cherry radish is constant, so the F-value, p-value, and letter labels are not applicable.*

**Table S3.** Physiological endpoints of Chinese cabbage under varying concentrations of PS-MPs.

| Treatment | Root biomass (mg) | Total chlorophyll content (mg/g) |
|---|---|---|
| Control | $59.00 \pm 14.73^{ab}$ | $1.30 \pm 0.15^{a}$ |
| 10mg/kg | $22.67 \pm 6.51^{b}$ | $1.16 \pm 0.20^{ab}$ |
| 50mg/kg | $37.00 \pm 15.13^{b}$ | $0.95 \pm 0.17^{b}$ |
| 100mg/kg | $87.00 \pm 31.75^{a}$ | $1.04 \pm 0.31^{ab}$ |
| *F* | 6.304 | 2.492 |
| *p* | 0.017 | 0.097 |

*[1] Results are presented as mean ± SD (Standard Deviation), N = 5.*

*[2] F-value: Indicates the ratio of between-group variance to within-group variance, assessing whether there are significant differences between groups.*

*[3] p-value: Reflects the statistical significance of the observed differences; a smaller p-value suggests significant differences.*

*[4] Letter labels (e.g., a, b, ab) were applied using Duncan's post hoc test to indicate specific group differences. Groups with the same letter are not significantly different, whereas those with different letters exhibit significant differences.*

**Table S4.** Physiological endpoints of cherry radish under varying concentrations of PS-MPs.

| Treatment | Root biomass (mg) | Total chlorophyll content (mg/g) |
|---|---|---|
| Control | 28.67±9.07$^{A}$ | 1.93±0.32$^{A}$ |
| 10mg/kg | 13.67±2.08$^{B}$ | 1.89±0.23$^{A}$ |
| 50mg/kg | 9.67±1.15$^{B}$ | 1.51±0.18$^{B}$ |
| 100mg/kg | 10.00±1.73$^{B}$ | 1.51±0.14$^{B}$ |
| *F* | 10.593 | 5.351 |
| *p* | 0.004 | 0.010 |

*[1] Results are presented as mean ± SD (Standard Deviation), N = 5.*

*[2] F-value: Indicates the ratio of between-group variance to within-group variance, assessing whether there are significant differences between groups.*

*[3] p-value: Reflects the statistical significance of the observed differences; a smaller p-value suggests significant differences.*

*[4] Letter labels (e.g., A, B, AB) were applied using Duncan's post hoc test to indicate specific group differences. Groups with the same letter are not significantly different, whereas those with different letters exhibit significant differences.*

**Table S5.** Biochemical endpoints of Chinese cabbage under varying concentrations of PS-MPs.

| Treatment | MDA content (nmol/g) | SOD activity (U/mgprot) | GSH content (mgGSH/gprot) | DPPH free scavenging rate (%) |
|---|---|---|---|---|
| Control | 50.99±6.72[a] | 70.70±13.59[a] | 4.73±1.18[a] | 46.15±4.97[b] |
| 10mg/kg | 51.18±9.21[a] | 62.00±18.89[a] | 4.39±18.89[a] | 50.71±8.02[ab] |
| 50mg/kg | 46.64±16.48[a] | 49.92±13.66[a] | 3.68±13.66[a] | 68.31±9.25[a] |
| 100mg/kg | 48.66±6.62[a] | 44.67±22.43[a] | 3.43±22.43[a] | 44.88±8.04[b] |
| *F* | 0.124 | 1.348 | 1.130 | 5.877 |
| *p* | 0.943 | 0.326 | 0.393 | 0.020 |

*[1] Results are presented as mean ± SD (Standard Deviation), N = 5.*

*[2] F-value: Indicates the ratio of between-group variance to within-group variance, assessing whether there are significant differences between groups.*

*[3] p-value: Reflects the statistical significance of the observed differences; a smaller p-value suggests significant differences.*

*[4] Letter labels (e.g., a, b, ab) were applied using Duncan's post hoc test to indicate specific group differences. Groups with the same letter are not significantly different, whereas those with different letters exhibit significant differences.*

**Table S6.** Biochemical endpoints of cherry radish under varying concentrations of PS-MPs.

| Treatment | MDA content (nmol/g) | SOD activity (U/mgprot) | GSH content (mgGSH/gprot) | DPPH free scavenging rate (%) |
|---|---|---|---|---|
| Control | 76.89±19.58$^{AB}$ | 74.16±7.12$^{A}$ | 6.51±1.46$^{B}$ | 67.98±12.49$^{A}$ |
| 10mg/kg | 51.55±12.61$^{AB}$ | 78.83±10.73$^{A}$ | 2.79±0.95$^{C}$ | 67.93±20.31$^{A}$ |
| 50mg/kg | 84.33±33.34$^{A}$ | 50.28±26.49$^{AB}$ | 9.95±3.09$^{A}$ | 57.14±15.01$^{A}$ |
| 100mg/kg | 40.78±3.99$^{B}$ | 21.22±12.73$^{B}$ | 5.11±0.81$^{BC}$ | 69.02±7.90$^{A}$ |
| $F$ | 3.047 | 8.133 | 8.155 | 0.441 |
| $p$ | 0.092 | 0.008 | 0.008 | 0.730 |

*[1] Results are presented as mean ± SD (Standard Deviation), N = 5.*

*[2] F-value: Indicates the ratio of between-group variance to within-group variance, assessing whether there are significant differences between groups.*

*[3] p-value: Reflects the statistical significance of the observed differences; a smaller p-value suggests significant differences.*

*[4] Letter labels (e.g., A, B, AB) were applied using Duncan's post hoc test to indicate specific group differences. Groups with the same letter are not significantly different, whereas those with different letters exhibit significant differences.*
